# Characterization of phenotypic variation and genome aberrations observed among *Phytophthora ramorum* isolates from diverse hosts

**DOI:** 10.1186/s12864-018-4709-7

**Published:** 2018-05-02

**Authors:** Marianne Elliott, Jennifer Yuzon, Mathu Malar C, Sucheta Tripathy, Mai Bui, Gary A. Chastagner, Katie Coats, David M. Rizzo, Matteo Garbelotto, Takao Kasuga

**Affiliations:** 1 Washington State University Puyallup Research and Extension Center, Puyallup, Washington 98371 USA; 2 Department of Plant Pathology, University of California, Davis, California 95616 USA; 3 Computational Genomics Lab, Structural Biology and Bioinformatics Division, CSIR Indian Institute of Chemical Biology, Kolkata, 700032 India; 4 Crops Pathology and Genetics Research Unit, USDA Agricultural Research Service, Davis, California 95616 USA; 5 Department of Environmental Science, Policy, and Management, University of California, Berkeley, California 94720 USA

**Keywords:** Invasive pathogens, Aneuploidy, Transposable elements, Loss of heterozygosity

## Abstract

**Background:**

Accumulating evidence suggests that genome plasticity allows filamentous plant pathogens to adapt to changing environments. Recently, the generalist plant pathogen *Phytophthora ramorum* has been documented to undergo irreversible phenotypic alterations accompanied by chromosomal aberrations when infecting trunks of mature oak trees (genus *Quercus*)*.* In contrast, genomes and phenotypes of the pathogen derived from the foliage of California bay (*Umbellularia californica*) are usually stable. We define this phenomenon as host-induced phenotypic diversification (HIPD). *P. ramorum* also causes a severe foliar blight in some ornamental plants such as *Rhododendron* spp. and *Viburnum* spp., and isolates from these hosts occasionally show phenotypes resembling those from oak trunks that carry chromosomal aberrations. The aim of this study was to investigate variations in phenotypes and genomes of *P. ramorum* isolates from non-oak hosts and substrates to determine whether HIPD changes may be equivalent to those among isolates from oaks.

**Results:**

We analyzed genomes of diverse non-oak isolates including those taken from foliage of *Rhododendron* and other ornamental plants, as well as from natural host species, soil, and water. Isolates recovered from artificially inoculated oak logs were also examined. We identified diverse chromosomal aberrations including copy neutral loss of heterozygosity (cnLOH) and aneuploidy in isolates from non-oak hosts. Most identified aberrations in non-oak hosts were also common among oak isolates; however, trisomy, a frequent type of chromosomal aberration in oak isolates was not observed in isolates from *Rhododendron*.

**Conclusion:**

This work cross-examined phenotypic variation and chromosomal aberrations in *P. ramorum* isolates from oak and non-oak hosts and substrates. The results suggest that HIPD comparable to that occurring in oak hosts occurs in non-oak environments such as in *Rhododendron* leaves. *Rhododendron* leaves are more easily available than mature oak stems and thus can potentially serve as a model host for the investigation of HIPD, the newly described plant-pathogen interaction.

**Electronic supplementary material:**

The online version of this article (10.1186/s12864-018-4709-7) contains supplementary material, which is available to authorized users.

## Background

The underlying genetic mechanisms of how invasive pathogens adapt to new hosts during co-evolutionary interactions is not well understood. In some systems, initial phenotypic plasticity or depauperate interactions occur followed by adaptation to new environments [1–3].

*Phytophthora ramorum* is a recent example of an invasive pathogen attacking more than 130 host plant species across 75 genera [4]. It causes Sudden Oak Death (SOD) or Sudden Larch Death (SLD) [5] in forests, and Ramorum blight on most of its other hosts including ornamentals in nurseries. As of now, four clonal lineages of *P. ramorum* have been described, and all are believed to be exotic to Europe and North America. Epidemiological and regulatory consequences of the disease have caused millions of dollars in losses to ornamental nurseries, larch plantations, and native vegetation [6, 7].

The NA1 clonal lineage of *P. ramorum* is predominant in natural ecosystems and production nurseries in the Pacific Coast region of North America. Despite its primary mode of asexual reproduction and the lack of sexual recombination [8], the pathogen shows diverse colony morphology and aggressiveness. In culture, for instance, wild type (*wt*) *P. ramorum* grows as a uniform, roughly circular colony mostly appressed to the culture media [9]. However, some isolates exhibit a range of phenotypes such as irregular colony shape, and unstable or slower growth rate, which we refer to as non-wild type (*nwt*). Additionally, some *nwt* isolates become senescent, i.e. they cease to grow upon subculturing, whereas other show greater aerial growth. Inoculation experiments have shown that isolates with *nwt* morphology are less aggressive than *wt* isolates [9–11]. Among isolates from natural forests, *nwt* morphology is associated with isolates from oak species (genus *Quercus*), and *nwt* isolates are rare among California bay (*Umbellularia californica*). California bay is an epidemiologically important foliar host, due to the abundant sporulation of the pathogen it supports on its leaves.

We have previously demonstrated that the *nwt* phenotype can experimentally be generated [12]. When *wt P. ramorum* isolates from California bay were inoculated into and re-isolated from mature canyon live oak (*Q. chrysolepsis*) and Shreve oak (*Q. parvula* var. *shrevei*), a large percentage (40–60%) of re-isolates were found to display *nwt* phenotype and chromosomal alterations such as monosomy, trisomy, and copy number neutral loss of heterozygosity (cnLOH), which were accompanied by de-repression of diverse transposable elements (TEs). Because the *wt*/*nwt* variation does not associate with population genetic markers, and can be induced by inoculation onto *Quercus*, the concept of host-induced phenotypic diversification (HIPD) was formulated [11, 12]. *Nwt* phenotype has also been observed among isolates derived from Lawson cypress (*Chamaecyparis lawsoniana*), which belonged to EU1, the dominant clonal lineage in Europe. A dead-end host is one in which the pathogen does not produce the infectious stage [13]. Both oaks and Lawson cypress are considered to be dead-end hosts; therefore, the epidemiological significance of *nwt* phenotype is not clear [12].

It is worth noting that Sudden Oak Death is actually not “sudden” at all. In some cases, individual oak trees infected with *P. ramorum* do not die rapidly. Some live for many years, and in others, the host can recover from the disease [14]. Such oak survival may be associated with the conversion of *P. ramorum* to slow growing *nwt*, allowing the host to contain the pathogen, or the pathogen may become senescent and therefore may be effectively eradicated [12]. Colony instability, slower growth rates, and lower aggressiveness of *nwt* phenotypes can be viewed as an undescribed type of host-pathogen interaction, and generation of the *nwt* phenotype may be a consequence of an active plant defense. Alternatively, genome rearrangements such as formation of aneuploids and loss of heterozygosity, and the associated phenotypic changes can be viewed as a part of an evolutionary process in which genome diversification contributes to the adaptation of the invasive pathogen to various new and unfamiliar environmental situations [15]. In addition, de-repression of TEs observed among *nwt* isolates [11, 12] has evolutionary implications as stress-induced changes in TE activity are claimed to promote structural variation in the genome and facilitate rapid adaptation in invasive species [16]. In either case, the study of HIPD will provide us insights into host-pathogen interaction in which coevolutionary history is not present, and further study on HIPD may lead us to the development of novel strategies to manage the pathogen.

Inoculation of mature oak trees to unravel the genetic mechanisms underlying host-induced chromosomal alterations is not practical for many reasons, including availability of specimens, strict regulations of field experiments involving infectious agents as well as heterogeneity of biotic and abiotic environments among specimens. However, the *nwt* phenotype has occasionally also been observed in *P. ramorum* derived from infectious hosts such as *Rhododendron* spp. in production nurseries [10]. Due to its size and availability, use of *Rhododendron* for inoculation studies has a significant advantage over oak. It is not yet clear whether *nwt* phenotypes observed in isolates from *Rhododendron* and other non-oak hosts and those from oaks are driven by the same genetic mechanism. The objective of this research was to characterize phenotypic and genomic variations among *P. ramorum* isolates from the foliage of ornamental plants and other substrates and compare them to those found in oak isolates. Taking advantage of a significantly improved version of *P. ramorum* genome recently assembled using PacBio sequencing, we analyzed genome alterations at high resolution. We will then discuss the implications for a general genetic mechanism that generates large mutational events to the evolution of an invasive pathogen.

## Results

### Phenotypic analysis of *P. ramorum* isolates from foliar hosts

A collection of 110 isolates of *Phytophthora ramorum* all belonging to the NA1 lineage was collected between 2006 and 2015 in Washington State. A total of 81 isolates were from production nurseries, while the remaining 29 were from natural streams or landscape plantings. A subset of 30 isolates from this collection was subjected to further examination, including eight *nwt* and 22 *wt i*solates. Four isolates from California, Pr-102 (*nwt*, oak), Pr-106 (*wt,* bay), CDFA1644994–2 (*wt*, *Camellia*), and ND886 (*wt*, *Camellia*) were also included in the analysis. This brings to 34 the total number of isolates subjected to an in depth analysis (Washington State University Collection, WA 2017 in Table 1 and Additional file 1).

**Table 1:** Isolates used in this study

Isolate numbers	Source^a^	Year	State	SSR multilocus genotype	Colony phenotype	Contact
Washington State University Collection (WA 2017)						
WSU106–0009	Stream bait	2006	WA	NA1-D02	*nwt*	M. Elliott & G. Chastagner
WSU106–0019 ^b^	*Phoradendron serotinum subsp. macrophyllum*	2006	WA	NA1-D06	*wt*	M. Elliott & G. Chastagner
WSU107–0016	Soil bait	2007	WA	NA1-D01	*wt*	M. Elliott & G. Chastagner
WSU107–0019	*Rhododendron* sp.	2007	WA	NA1-A21	*nwt*	M. Elliott & G. Chastagner
WSU107–0042	*Rhododendron* sp.	2007	WA	NA1-A21	*nwt*	M. Elliott & G. Chastagner
WSU107–0043	*Rhododendron* sp.	2007	WA	NA1-A21	*nwt*	M. Elliott & G. Chastagner
WSU107–0054	*Rhododendron* sp.	2007	WA	NA1-A03	*wt*	M. Elliott & G. Chastagner
WSU107–0057 ^b^	*Rhododendron* sp.	2007	WA	NA1-A03	*wt*	M. Elliott & G. Chastagner
WSU107–0066	*Rhododendron* sp.	2007	WA	NA1-D01	*wt*	M. Elliott & G. Chastagner
WSU107–0072 ^b^	*Rhododendron* sp.	2007	WA	NA1-A05	*nwt*	M. Elliott & G. Chastagner
WSU107–0073	*Rhododendron* sp.	2007	WA	NA1-A05	*wt*	M. Elliott & G. Chastagner
WSU107–0081 ^b^	*Arbutus unedo*	2007	WA	NA1-A25	*wt*	M. Elliott & G. Chastagner
WSU107–0086 ^b^	*Rhododendron* sp.	2007	WA	NA1-A03	*wt*	M. Elliott & G. Chastagner
WSU107–0093	*Rhododendron* sp.	2007	WA	NA1-D02	*wt*	M. Elliott & G. Chastagner
WSU107–0094	*Rhododendron* sp.	2007	WA	NA1-A03	*nwt*	M. Elliott & G. Chastagner
WSU107–0095	*Rhododendron* sp.	2007	WA	NA1-A10	*wt*	M. Elliott & G. Chastagner
WSU107–0096	*Rhododendron* sp.	2007	WA	NA1-A10	*wt*	M. Elliott & G. Chastagner
WSU107–0100	*Rhododendron* sp.	2007	WA	NA1-A29	*wt*	M. Elliott & G. Chastagner
WSU108–0003	Stream bait	2008	WA	NA1-A28	*wt*	M. Elliott & G. Chastagner
WSU108–0006	*Viburnum tinus*	2008	WA	NA1-A17	*wt*	M. Elliott & G. Chastagner
WSU108–0021 ^b^	*Viburnum tinus*	2008	WA	NA1-A02	*wt*	M. Elliott & G. Chastagner
WSU108–0022	*Viburnum tinus*	2008	WA	NA1-A02	*wt*	M. Elliott & G. Chastagner
WSU108–0024	*Viburnum tinus*	2008	WA	NA1-A02	*wt*	M. Elliott & G. Chastagner
WSU108–0025	*Viburnum tinus*	2008	WA	NA1-A02	*wt*	M. Elliott & G. Chastagner
WSU111–0001 ^b^	Stream bait	2011	WA	NA1-A29	*wt*	M. Elliott & G. Chastagner
WSU111–0002	Stream bait	2011	WA	NA1-A05	*wt*	M. Elliott & G. Chastagner
WSU115–0077	*Rhododendron* sp.	2015	WA	NA1-A13	*nwt*	M. Elliott & G. Chastagner
WSU115–0089	Soil bait	2015	WA	NA1-A17	*wt*, scenes	M. Elliott & G. Chastagner
WSU115–0095	Soil bait	2015	WA	NA1-A30	*wt*	M. Elliott & G. Chastagner
WSU115–0118 ^b^	*Rhododendron* sp.	2015	WA	NA1-A13	*nwt*, scenes	M. Elliott & G. Chastagner
UC Davis Collection (CA 2017)						
BS2014–584	*Notholithocarpus densiflorus*	2014	CA		*nwt*	D. Rizzo
BS96	*Umbellularia californica*	2004	CA		*nwt*	D. Huberli
Pr-106 ^b^	*Umbellularia californica*	2001	CA		*wt*	D. Rizzo
Pr-218	*Rhamnus cathartica*	2002	CA		*wt*	D. Rizzo
Pr-438	*Arbutus menziesii*	2006	CA		*wt*	D. Rizzo
Pr-451	*Sequoia sempervirens*	2004	CA		*wt*	D. Rizzo
Pr-455	*Osmorhiza berteroi*	2005	CA		*nwt*	D. Rizzo
Pr-458	*Adiantum jordanii*	2005	CA		*wt*	D. Rizzo
Pr-467	*Corylus cornuta*	2006	CA		*wt*	D. Rizzo
Pr-472	*Choisya ternata*	2006	CA		*nwt*	D. Rizzo
Pr-486	*Camellia* sp.	2006	CA		*nwt*	D. Rizzo
Pr-1537	*Abies grandis*	2012	CA		*wt*	D. Rizzo
Pr-1652	Stream bait	2014	CA		*wt*	D. Rizzo
9D1	(Re) Pr-1556 from *Q. agrifolia* log	2012	CA		*wt*	T. Kasuga
California Department of Food and Agriculture (CDFA) Collection (CA 2017)						
CDFA1418886; ND886 ^b^, ^c^	*Camellia* sp.	2004	CA	NA1-B20	*wt*	S. Latham & C. Blomquist
CDFA1644994–2	*Camellia* sp.	2011	CA		*wt*	S. Latham & C. Blomquist
UC Davis Collection, genome analysis published (Kasuga et al. [12]) (CA 2016)						
Pr-16	*Quercus agrifolia*	2000	CA		*nwt*	D. Rizzo
Pr-102 ^b^	*Quercus agrifolia*	2001	CA		*nwt*	D. Rizzo
Pr-745	Rain water near infected *U. californica*	2010	CA		*wt*	Phytosphere Research
Pr-745#3	(Re) Pr-745 from *Q. chrysolepis*	2011	CA		*nwt*	K. Aram
Pr-745#4	(Re) Pr-745 from *Q. chrysolepis*	2011	CA		*nwt*	K. Aram
Pr-1556	*Umbellularia californica*	2011	CA		*wt*	T. Kasuga
Pr-1556#7#1	(Re) Pr-1556 from *Q. chrysolepis* & race tube	2013	CA		*nwt*	T. Kasuga
Pr-140.7	*Quercus parvula* var. *shrevei*	2014	CA		*nwt*	Phytosphere Research
Pr-140.9	*Quercus parvula* var. *shrevei*	2014	CA		*wt*	Phytosphere Research
MK516a	*Quercus agrifolia*	2008	CA		*nwt*	M. Garbelotto

^a^ (Re) indicates re-isolates. These isolates were artificially inoculated and recovered from indicated hosts

^b^ Isolates that have been both sequenced and phenotyped

^c^ CDFA1418886 is abbreviated to ND886 in this report

**Fig. 1:**
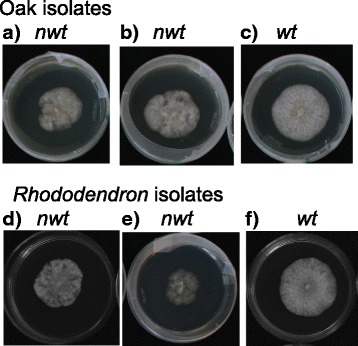
*Nwt* isolates from foliage of *Rhododendron* plants are indistinguishable from those from oak stem lesions. Isolates were grown on 1× CV8A medium for 1 week in dark. **a**, **b** *nwt* isolates from coast live oak showing irregular colony morphology, whereas **c** *wt* isolate from coast live oak grew to a circular mycelial mat. **d**, **e** *nwt* isolates from *Rhododendron* and **f** *wt* isolate from *Rhododendron*

Two of the 34 isolates, namely WSU115–0089 (*wt*, soil) and WSU115–0118 (*nwt*, *Rhododendron*) stopped growing when subcultured. This is a commonly observed phenomenon termed early senescence and often seen among isolates with *nwt* phenotype [11]. Importantly, *nwt* colony morphology observed among isolates from *Rhododendron* and a stream in Washington State is indistinguishable from those observed among isolates from oaks in California (Fig. 1 and Additional file 2).

**Fig. 2:**
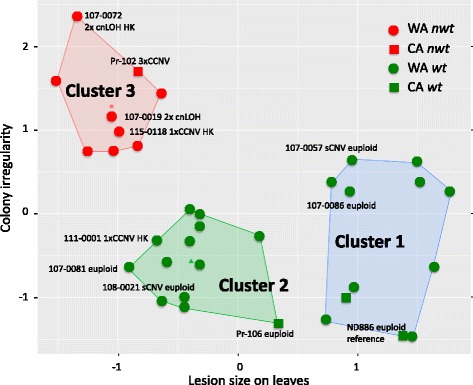
Phenotypic profiles of *Phytophthora ramorum* isolates. Each dot represents the standardized mean value of one of 34 isolates for which irregularity index (percent difference of the maximum and minimum radii within a 45-degree sector) of axenic culture and lesion size on *Rhododendron* foliage were measured. K-means clustering grouped isolates into three clusters. Isolates with *nwt* colony phenotype (shown in red circles and squares) are found only in Cluster 3. Cluster 1 contains *wt* isolates that were aggressive on *Rhododendron* leaves, whereas Cluster 2 contains isolates that were less aggressive and had wild-type colony morphology. Symbols are: red circle, *nwt* Washington state isolates; red square, *nwt* California isolates; green circle, *wt* Washington State isolates; green square, *wt* California isolates. Isolate numbers and chromosomal aberration types are indicated for those subjected to genome analyses

**Fig. 3:**
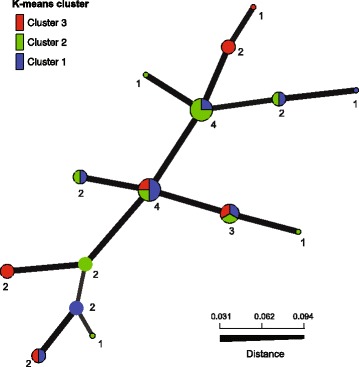
Minimum spanning network of 15 SSR MLGs observed among the 30 Washington State isolates. Node colors represent K-means cluster membership proportional to the pie size and the number of *P. ramorum* isolates is indicated at each node. Edge thickness represents minimum genetic distance between genotypes. Population subdivision according to the K-means clusters was not detected

Here we introduce the “irregularity index,” defined as the maximum percent deviation of the maximum and minimum radii within a 45-degree sector of the colony (Additional file 3), and used as a quantitative measure of the irregularity of colony morphology. In addition, aggressiveness of isolates was evaluated on detached *Rhododendron* leaves [10]. K-means cluster analysis identified three distinctive groups using the combination of irregularity index and lesion size (Fig. 2). All eight *nwt* isolates from Washington State were unequivocally found in Cluster 3, a group characterized by high irregularity index and reduced aggressiveness. Values for irregularity index and relative lesion area of isolates in Cluster 3 were significantly different from those in Clusters 1 and 2 (*p* < 0.001 for both, 1-way ANOVA, Tukey-Kramer multiple comparisons, Additional file 4). In contrast, Clusters 1 and 2 comprised exclusively *wt* isolates having low irregularity index. Isolates in Clusters 1 and 2 were indistinguishable by their growth rates on an artificial medium (t-test, *p* = 0.58) or irregularity index (t-test, *p* = 0.34), however, Cluster 2 isolates showed significantly smaller lesion sizes on *Rhododendron* leaves than Cluster 1 isolates (t-test, *p* = 5.9 × 10^− 11^). With regards to California isolates, the trisomic *nwt* isolate Pr-102 (used for whole genome Sanger sequencing in 2006 [17]) was found in Cluster 3, whereas two and one *wt* isolates were found in Cluster 1 and Cluster 2, respectively. When the 30 Washington State isolates were analyzed using eight simple sequence repeat (SSR) markers, 15 unique multilocus genotypes (MLGs) were identified (Fig. 3, Additional file 5). Multilocus SSR markers, however, failed to show genetic differentiation among isolates in the three K-means clusters (AMOVA, *p* = 0.4). In fact, six SSR MLGs were shared between K-means clusters. This result indicates that population subdivision is not attributable to the observed K-means clusters. The result rather indicates that pathogen individuals can independently and rapidly change in cluster membership based on phenotype.

**Fig. 4:**
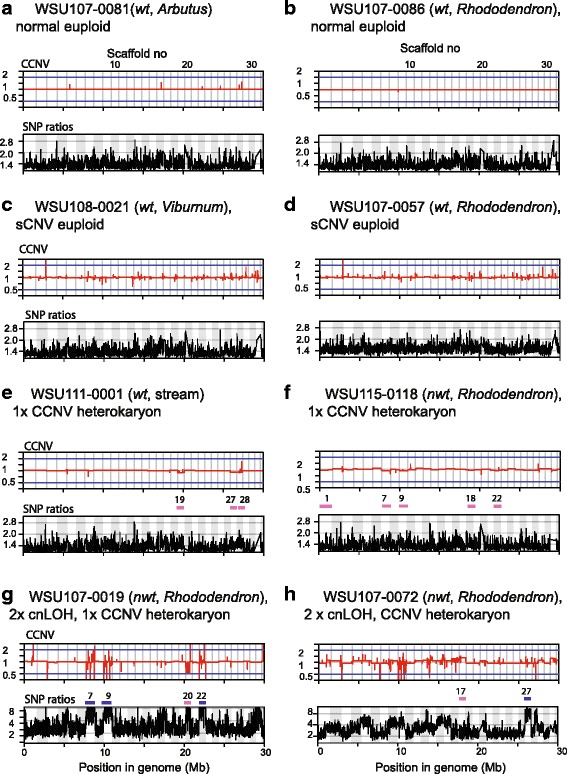
Read-depth analyses revealed diverse chromosomal aberrations *in P. ramorum* from Washington State isolates. BIC-seq analysis reveals CCNVs (upper graph for each panel), while a read-depth analysis for heterozygous allele ratios (SNP ratios) detects LOH (lower graph). A concatenated view of the 31 largest scaffolds with a total length of 30 MB (c.a. half of the genome) is shown. A wild type euploid isolate ND886 was used as a reference. Scaffold numbers for large CCNV regions are indicated with pink bars, and those for cnLOH are shown with blue bars. Scales show log (base 2) fold difference between sample isolates and the reference isolate for BIC-seq analysis and log (base 2) ratios of SNP alleles of sample isolates for the heterozygous allele frequency analysis. **a** WSU107–0081 and **b** WSU107–0086 are examples of CCNV profile for the category “normal euploid”. Two *wt* isolates, **c** WSU108–0021 from *Viburnum* and **d** WSU107–0057 from *Rhododendron,* showed numerous CNVs in small chromosomal segments (sCNV euploid). **e** *Wt* stream isolate WSU111–0001 showed c.a. 10% reduction in copy numbers in three scaffolds 19, 27, and 28, indicating it is a heterokaryon of monosomy (1× CCNV) and euploid. **f** *Nwt Rhododendron* isolate WSU115–0118 showed slight copy number changes in numbers of scaffolds, indicating it is a mixture of monosomic nuclei. **g** *Nwt Rhododendron* isolate WSU107–0019 showed increases in SNP ratios in scaffold 7, 9, and 22 while a consistent change in chromosome copy number was not detected, which indicates cnLOH. Close inspection of short segments with CNV seen as spikes in cnLOH regions (above the blue bars) reveal the *wt* reference genome has heterozygous indels in these regions. Loss of chromosomal segments harboring these indels in the *nwt* isolate WSU107–0019 resulted in spikes in the BIC-seq analysis. **h** A *nwt* isolate WSU107–0072 revealed CCNVs and cnLOH indicating it is a heterokaryon having cnLOH and aneuploid nuclei

We performed genome analyses on 11 of the total 34 isolates that were phenotyped, eight from Washington State (WA) and three from California (CA) (Fig. 4, details described in the following section). Aneuploidy and loss of heterozygosity (LOH) were detected for one and four isolates in Clusters 2 and 3, respectively. On the other hand, euploid isolates (including one sCNV euploid individual discussed later) were never found in Cluster 3. The enrichment of aneuploid and LOH isolates in Cluster 3 in relation to Cluster 1 and 2 is statistically significant (Fisher exact test, *p* = 0.02).

When relative radial growth rate was included, it was not possible to differentiate groups [18] so this variable was dropped from the cluster analysis. Interestingly, no correlation was identified between relative radial growth rate and lesion size on *Rhododendron* (Pearson’s *r* = 0.16, *p* = 0.34). This suggests that in vitro radial growth does not correlate well with fitness of isolates. This result is not unexpected and has been described for a range of plant pathogens [19].

The occurrence of *nwt* among isolates from *Rhododendron* plants in Washington State (seven out of 35 NA1 isolates collected in Washington State between 2006 and 2015) is significantly more frequent than that observed among California bay in California (two *nwt* out of 152 California bay isolates, Fisher’s exact test, *p* = 5.8 × 10^− 4^). However, independence of phenotypic changes is harder to prove when dealing with ornamental plants, due to the fact that plants are often traded among facilities.

The high frequency of *nwt* isolates from *Rhododendron* from Washington State motivated us to examine the phenotype of 39 nursery isolates from California. All 39 isolates (7 from *Rhododendron* spp.) were obtained from California production nurseries between 2011 and 2015, and all showed *wt* colony morphology (Additional file 1). However, the frequencies of *nwt* isolates from *Rhododendron* in Washington State (7 in 35) and that in California (0 in 7) are not statistically significant (Fisher’s exact test, *p* = 0.33). Lack of significant differences is due to uneven sampling of plants from the two States.

### Analysis of copy number variations (CNVs)

It has been shown that oaks but not bay induce genomic alterations that are tightly associated with *nwt* colony phenotype [12]. The host mechanism triggering such changes is unknown, but phenolic compounds in the oak bark are suspected to be the cause of such alterations. This raises the question of whether compounds in leaves of *Rhododendron* or in other hosts and substrates may also induce genomic aberrations in *P. ramorum*.

A representative sample of 11 isolates (*wt* and *nwt*, from different hosts and belonging to all three K-means clusters, see Table 1) was used to address this issue. The selected set of isolates was sequenced and analyses of copy number variations (CNVs) and heterozygous allele ratios were performed using an updated assembly of the *P. ramorum* genome (ND886 V1). The current version of the assembly in fact contains 295 scaffolds, a significant improvement from the previous assembly of 2576 scaffolds [17]. CNV analysis can detect changes in copy number of genome segments (≥100 bp) by deletion or duplication, whereas the analysis of heterozygous allele ratio (SNP ratio) can detect copy number changes as well as copy-neutral loss of heterozygosity (cnLOH), which cannot be detected by the CNV analysis. We have previously characterized genome aberrations in isolates mainly from oaks (UC Davis Collection, genome analysis published, CA 2016 in Tables 1 and 2 [12]). In order to discern host-dependent patterns of genome aberration, genomes of additional 12 isolates from rare non-oak host species in California were sequenced (UC Davis Collection, CA 2017 in Tables 1 and 2). To investigate whether oak logs as opposed to live trees can induce genome aberrations, ten re-isolates recovered from logs of three oak species previously inoculated with isolates from California bay were also included for the genome analysis (UC Davis Collection, CA 2017 in Table 1 and Additional file 1). In total, 32 isolates (Additional file 1; eight from Washington State University Collection, WA 2017; 23 from UC Davis Collection, CA2017; and one from CDFA Isolates, CA 2017) underwent CNV and SNP ratio analyses (Table 2 and Additional file 1).

**Table 2:** Summary of isolates with chromosomal aberrations

CCNV category ^a^	Isolate ^b^	colony phenotype	Source ^c^
1× CCNV	Pr-140.9 (*wt*, QUPS)	*wt*	CA 2016
1× CCNV HK	WSU111–0001 (*wt*, stream)	*wt*	WA 2017
1× CCNV complex HK	Pr-140.7 (*nwt*, QUPS)	*nwt*	CA 2016
1× CCNV HK	Pr-1556#7#1 (*nwt*, bay→QUCH→race tube)	*nwt*	CA 2016
1× CCNV HK	WSU115–0118 (*nwt*, *Rhododendron*)	*nwt*	WA 2017
3× CCNV + 1× CCNV Complex HK	BS2014–584 (*nwt*, tanoak canker)	*nwt*	CA 2017
2× cnLOH	MK516a (*nwt*, QUAG)	*nwt*	CA 2016
2× cnLOH	WSU107–0019 (*nwt, Rhododendron*)	*nwt*	WA 2017
2× cnLOH	Pr-455 (*nwt*, *Osmorhiza*)	*nwt*	CA 2017
2× cnLOH complex HK	WSU107–0072 (*nwt*, *Rhododendron*)	*nwt*	WA 2017
3× CCNV	Pr-745#3 (*nwt*, bay→QUCH)	*nwt*	CA 2016
3× CCNV	Pr-218 (*wt, Rhamnus*)	*wt*	CA 2017
3× CCNV	Pr-102 (*nwt*, QUAG)	*nwt*	CA 2016
3× CCNV + 1× CCNV HK	Pr-745#4 (*nwt*, bay→QUCH)	*nwt*	CA 2016
3× CCNV + 2× cnLOH	Pr-16 (*nwt*, QUAG)	*nwt*	CA 2016
3× CCNV HK	BS96 (*nwt*, bay)	*nwt*	CA 2017
3× CCNV HK	Pr-472 (*Corylus*)	*nwt*	CA 2017
3× CCNV HK	Pr-486 (*Camellia*)	*nwt*	CA 2017
3× CCNV HK	9D1 (*wt*, bay→QUAG log)	*wt*	CA 2017
sCNV euploid	WSU108–0021 (*wt*, *Viburnum*)	*wt*	WA 2017
sCNV euploid	WSU107–0057 (*wt*, *Rhododendron*)	*wt*	WA 2017

^a^ Due to CNV and allele ratio analyses, chromosomal aberrations were categorized into five groups: 1× CCNV, monosomy; 3× CCNV, trisomy; cnLOH, copy-number neutral loss of heterozygosity, and short CNV (sCNV) euploid. HK indicates heterokaryotic isolate

^b^ In the parentheses, colony phenotypes and host plants are indicated. Arrows such as bay→QUCH indicates the isolate was originally from California bay, inoculated and recovered from canyon live oak (see Kasuga et al., [12])

Species names are: bay (California bay, *Umbellularia californica*); QUAG (coast live oak, *Q. agrifolia*); QUEN (Engelmann oak, *Q. engelmannii*); QUWI (interior live oak, *Q. wislizeni*); QUCH (canyon live oak, *Q. chrysolepis*); QUPS (Shreve oak, *Q. parvula* var. *shrevei*)

^c^ CA 2016 indicates California isolates, published in Kasuga et al., [12]. CA 2017 and WA 2017 indicate California and Washington State isolates, respectively, determined in this study

#### Genomes of the *wt* isolates

Two isolates with *wt* culture morphology, WSU107–0081 (*Arbutus*, Cluster 2) and WSU107-0086 (*Rhododendron*, Cluster 1), showed mostly linear lines across the concatenated scaffolds when referenced to the *wt* euploid isolate ND886 (*Camellia*, Cluster 1) (shown in red in Fig. 4a, b). This indicates isolates WSU107–0081 and WSU107–0086 are also euploids carrying minimal genome alterations. Average heterozygous allele ratios (SNP ratios), using a 10 KB long non-overlapping sliding window, were close to one showing that the two *wt* isolates have a balanced set of chromosomes. On the other hand, Cluster 2 *wt* isolate WSU108-0021 from *Viburnum* (Fig. 4c) and Cluster 1 *wt* isolate WSU107-0057 from *Rhododendron* (Fig. 4d) showed a number of spikes along the concatenated scaffolds in the CNV analysis, indicating copy number variation in short segments. In these two isolates, gain or loss of numerous short segments, 100 to 10,000 bp in size, was found throughout the genome but other than that, no large CNVs or LOH spanning entire scaffolds were detected (scaffold boundaries indicated with vertical lines). This is a new type of chromosome aberration, which we termed short copy number variation (sCNV) euploid. Genomes of these two isolates were analyzed in further detail (see below).

Isolate WSU111-0001 (*wt*, Cluster 2) from a Washington stream showed a decrease in copy number throughout scaffolds 19, 27 and 28 (Fig. 4e), indicating a chromosome loss. Monosomy would result in a 50% chromosomal content change, but instead an approximate 10% decrease was observed across the three contigs. This indicates WSU111-0001 is a heterokaryon in which normal euploid nuclei outnumber monosomic nuclei 9 to 1. We suggest that the large ratio of euploid nuclei hid the manifestation of the aneuploid-driven *nwt* phenotype.

#### Genomes of the *nwt* isolates

Genome sequences of three out of the seven *nwt* isolates from *Rhododendron* were analyzed. All three *nwt* isolates had extensive CNVs and/or changes in average heterozygous allele ratios across the entirety of several scaffolds, indicating an occurrence of large chromosomal aberrations. These observations are consistent with previous findings regarding the tight association between *nwt* phenotype and the presence of large stretches (> 300 Kb) of CNVs [12]. One of the Cluster 3 *nwt* isolates, namely WSU115–00118, showed uneven copy number ratios in several scaffolds (e.g. 1, 7, and 9), indicating this isolate is a heterokaryon carrying a small number of nuclei that are monosomic at several chromosomes (Fig. 4f). In a *nwt* isolate WSU107–0019 from Cluster 3 instead, SNP ratio peaks detected along large stretches of genome were indicative of loss of heterozygosity (LOH), but chromosomal copy number changes in LOH regions (e.g. scaffolds 7, 9, and 22) were not detected (Fig. 4g). These LOH regions thus correspond to a chromosome aberration known as copy-neutral loss of heterozygosity (cnLOH). Short segments with CNV seen as spikes in cnLOH regions (scaffolds 7, 9, and 22) indicate heterozygous indels (> 100 bp). In the SSR analysis, the biallelic markers PrMS45 located at scaffold 9 produced a single PCR product supporting LOH at the chromosomal region (Additional file 5). Note that a partial monosomy at scaffold 20 was also detected. The third Cluster 3 *nwt* isolate WSU107–0072 from *Rhododendron* showed a complicated CNV pattern with increases in allele ratios in entire affected scaffolds (Fig. 4h). In addition, a signature for cnLOH is seen at scaffold 27. This indicates that this isolate is a heterokaryon containing aneuploid nuclei and nuclei with cnLOH.

In summary, three out of five *wt* isolates and all three *nwt* isolates were found to carry chromosomal aberrations. Two isolates showed numerous copy number variations in short segments, which is a new type of chromosomal aberration. These were analyzed in detail in the following section.

#### Analysis of genome aberrations in the short copy number variation (sCNV) euploid

Cluster 1 *wt* isolate WSU107–0057 from *Rhododendron* (Fig. 4d) and Cluster 2 *wt* isolate WSU108–0021 from *Viburnum* (Fig. 4c) showed copy number variation in short segments (shorter than 10 kb, a majority of fragments shorter than 1 kb), but otherwise remained euploid. Note that because these isolates were derived from different hosts in different production nurseries, these chromosomal aberrations were possibly generated independently. CNV analysis revealed both isolates carried copy number variations in comparable numbers and sizes of short segments in their genomes (the 5th and 6th columns in Table 3). Interestingly, the majority of the variable segments were shared between the two isolates (the last column in Table 3), implicating a common genetic mechanism underlies the chromosomal aberrations in these two isolates. In comparison to the three representative *wt* euploids, i.e. WSU107–0081 from *Arbutus*, WSU107–0086 from *Rhododendron*, and Pr-106 from bay (see also Additional file 6 G), the sCNV euploid isolates have three to six times more sCNV sites. Unlike the formation of monosomy or trisomy, in which copy number changes are 0.5× and 1.5×, respectively, sCNV euploids do not show discrete copy number changes (Fig. 4c, d). This may indicate that regions showing sCNV were comprised of multiple-copy DNA sequences, thus copy number changes estimated by the read depth analysis did not result in integer values.

**Table 3:** Short copy number variations are found in repetitive genomic regions

Isolates	WSU107–0081 (*wt*, Arbutus) normal euploid	WSU107–0086 (*wt, Rhododendron*) normal euploid	Pr-106 (*wt*, bay) normal euploid	WSU107–0057 (*wt, Rhododendron*) sCNV euploid	WSU108–0021 (*wt, Viburnum*) sCNV euploid	Overlap between WSU107–0057 and 108–0021
Total sCNV (average size)	71 (1888 bp)	71 (3021 bp)	102 (1751 bp)	357 (1255 bp)	446 (988 bp)	275
sCNV at multicopy regions	62 (87.3%)	68 (96%)	96 (94%)	319 (89.4%)	393 (88.1%)	267
sCNVs at TEs^a^	18 (25.3%)	6 (8.5%)	32 (31%)^a^	135 (37.8%)^a^	169 (37.9%)^a^	97
sCNVs at tandem repeat regions^a^	15 (21.5%)^a^	18 (25.3%)^a^	10 (9.8%)^a^	58 (16.2%)^a^	70 (15.7%)^a^	51

^a^ A randomization test showed that observed sCNVs at TEs or tandem repeats were significantly more frequent than expected by chance (*p* < 0.01)

Locations of sCNV in the genome were then examined. Approximately 30% of the genome of *P. ramorum* is comprised of multicopy regions (copy number ≥ 2, see Methods). Consistent with the continuous and wide range of copy number changes, 88–96% of sCNVs identified in the two sCNV euploid isolates, as well as those in the normal euploid isolates, were found in multicopy regions. Multicopy regions contain repetitive DNA, and eukaryotic repetitive DNA can be divided into two major categories: dispersed and tandem repeats (reviewed in [20, 21]). Dispersed repeats are typically transposable elements (TEs) occurring multiple times and widespread over the genome, whereas tandem repeats tend to be located in blocks at one or more locations on chromosomes. Repeat numbers of repetitive DNA sequences can readily be changed, in addition, there is mounting evidence repetitive DNA sequences may play an active role in genome arrangement processes [22, 23]. We located TEs and tandem repeats in the *P. ramorum* genome, and the co-localization between sCNVs and these repetitive DNA segments was evaluated. Over 11,000 transposable elements (TEs) belonging to diverse classes were predicted in the genome of *P. ramorum*, and 83% of them were also found in the multicopy regions. Approximately 38% of sCNV found in isolates WSU107–0057 and WSU108–0021 were found to overlap with predicted TEs (Table 3). Likewise, approximately 10,000 tandem repeats with repeat lengths ranging between 25 to 25,837 bp were identified in the *P. ramorum* genome of ND886. A total of 1578 tandem repeats were 100 bp or larger, and 16% of the sCNV in both sCNV euploid isolates were found to be co-localized with them. Co-localizations of sCNVs and tandem repeats were for the most part the same in both isolates (see last column in Table 3).

TEs and tandem repeats are ubiquitous in the *P. ramorum* genome and thus the observed overlaps of sCNV and repetitive DNA regions may simply occur by chance alone. When we randomized genomic locations of sCNV and evaluated frequencies of overlap, actual frequency of overlap was twice than that predicted by chance alone (*p* < 0.01, asterisks in Table 3), suggesting a biological association between sCNV and repetitive DNA regions.

#### Host dependency of chromosomal aberrations

It has been previously documented that the vast majority of *nwt* isolates in California have originated in oak stems, and CCNVs have previously been identified only in isolates from oaks. In this work, we analyzed a set of samples from California but not originally isolated from oaks. This set of samples included 5 *nwt* and 8 *wt* isolates from a range of hosts and substrates other than oak (see Table 1 for isolate numbers and hosts/substrates). Chromosomal aberrations were detected in all five *nwt* isolates and one of the eight *wt* isolates. We have also analyzed 10 *wt* re-isolates that were originally from California bay but that had been first inoculated in freshly cut logs from one of three oak species and re-isolated from them a month later (Additional file 1, UC Davis Collection, genomes analyzed, CA 2017). None of the re-isolates from oak logs changed their phenotype from *wt* to *nwt,* however, one of them showed chromosomal aberrations.

**Fig. 5:**
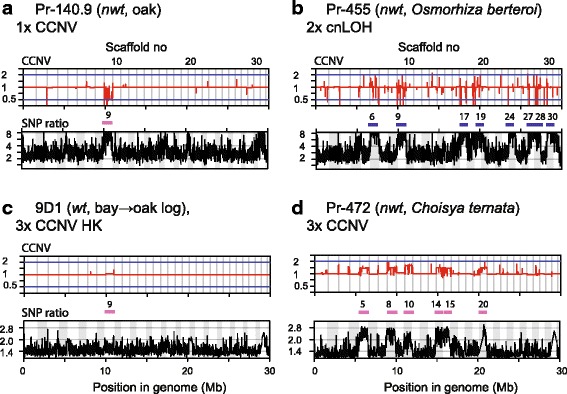
Examples of chromosomal aberrations found in *P. ramorum* in California. See Fig. 4 for further explanation of the figure legend. A wild type euploid isolate Pr-1556 was used as a reference. **a** *Nwt* isolate Pr140.9 from Shreve oak (*Q. parvula* var. *shrevei*) is an example of monosomy (1× CCNV) at scaffold 9. **b** *Nwt* isolate Pr-455 from mountain sweet cicely *Osmorhiza berteroi* showing copy number neutral LOH (cnLOH) at scaffolds 6, 9, 17, 19, 24, 27, 28, and 30. **c** A re-isolate 9D1 from a log of coast live oak showing a small copy number change at scaffold 9 in comparison to its original isolate Pr-1556 (*wt*, bay) used as a reference. 9D1 is likely a heterokaryon (HK) carrying trisomic (3× CCNV) and euploid nuclei. **d** *Nwt* isolate Pr-472 from Mexican orange blossom *Choisya ternata* is likely a heterokaryon carrying at least two types of trisomic nuclei, one with 3× CCNV at scaffolds (5, 8, 14, 15, 20) and the other with 3× CCNV at scaffold 10 (see Fig. 6 for scaffold linkage grouping)

Together with eight isolates with chromosomal aberrations previously identified from California [12] (marked as CA 2016 in Table 2 and Additional file 6), seven isolates from California examined in this study (CA 2017 in Table 2) and six isolates from Washington State (WA 2017 in Table 2), we have a total of 21 isolates carrying extensive chromosomal aberrations. All detected CNVs, except for those in the two sCNV euploid isolates, were large (> 700 Kb) and spanned entire scaffolds. These variations were thus referred as chromosomal copy number variations (chromosomal CNVs or CCNVs). Of the total of 21 isolates with aberrated genomes, six isolates are likely to contain full or partially monosomic nuclei (e.g. Fig. 5a, labeled as 1× CCNV), five contain copy number neutral LOH (Fig. 5b, cnLOH), and ten contain full or partially trisomic nuclei (Fig. 5c, d. 3× CCNV). Trisomic isolates were not identified among the Washington State isolates, and although the heterokaryotic isolate WSU107–0072 might contain a small number of trisomic nuclei, visible as CNVs at scaffold 17 (Fig. 4h), it was not included due to its ambiguity. The absence of trisomy is statistically significant when comparing California and Washington State isolates with nuclear aberrations, and when comparing WA isolates from rhododendrons and CA isolates from all hosts (Table 4, *p* = 0.01 CA *nwt* vs. WA *nwt*; *p* = 0.02 WA rhododendrons vs. CA all hosts). In conclusion, although further study is needed, chromosomal aberrations observed among isolates from *Rhododendron* in Washington State may be less likely to be trisomic than those from all hosts in California.

**Table 4:** Trisomy formation in *P. ramorum* isolates with nuclear aberrations, related to host and geographic location of origin

Comparison ^a^	Trisomic	Non-trisomic	*p*-value for the absence of trisomy ^b^
WA	0	6	0.011
CA	10	4	
WA *Rhododendron*	0	4	0.022
CA	10	4	

^a^ WA: Washington State isolates, CA: California isolates. Isolates passed through race tubes were not included

^b^ *P*-value according to Fisher’s exact test, 2-tailed

#### Chromosomal CNVs indicate physical linkages of scaffolds and preferential enrichment of haplotypes

The number of chromosomes in *P. ramorum* is not known, yet. However, judging from what is known about other *Phytophthora* species, *P. ramorum* probably carries five to 12 major chromosomes [24–26]. Our CNV analysis provides useful insights regarding the physical linkage of scaffolds, and thus it can potentially be used to anchor assembled genome scaffolds and to perform karyotype analysis.

**Fig. 6:**
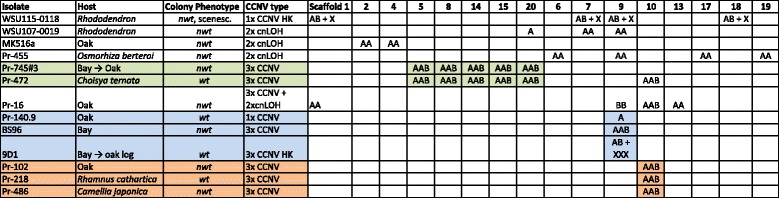
Summary of physical linkage of genome scaffolds and inferred haplotype combinations at each scaffold. Only the 20 largest scaffolds are shown and representative isolates carrying CCNVs and their scaffolds showing copy number variations were arranged to highlight physical linkage. See Additional file 7 for details. A and B denote homologous haplotypes at each individual scaffold. In other words, A on one scaffold does not necessarily link to A on the other scaffold. Inferred haplotype combinations are shown when those are deviated from a heterozygous disomic haplotype combination, AB. AAB, AA and A denote three copies of a DNA segment (i.e. trisomy), cnLOH, and copy deletion (i.e. monosomy), respectively. AB + X indicates a mixture of AB disomy and monosomy with an unknown haplotype. Likewise, AB + XXX indicates a mixture of AB disomy and trisomy with an unknown haplotype combination. HK in CCNV type indicates a heterokaryon. Scaffolds 5, 8, 14, 15, and 20 are likely physically linked, whereas each of scaffold 9 and 10 do not link to any of the 31 largest scaffolds in the current assembly

For example in isolates Pr-745#3, Pr-472, and BS2014-584 (Fig. 6 and Additional file 7, datasheet “31 Scaffolds”), five among the 20 largest scaffolds (scaffolds 5, 8, 14, 15, and 20) showed a 2.6× to 3× chromosomal copy number changes that is indicative of trisomy formation (3× CCNV). Note that a chromosomal copy number under 3 but over 2 is indicative of heterokaryotic hyphae carrying a variable ratio of trisomic and euploid nuclei [12]. In addition, owing to the incomplete genome assembly of *P. ramorum*, our CNV analysis cannot tell whether a full or partial trisomy is formed. Nevertheless, the concomitant and consistent increase in copy number in each isolate implicates that these scaffolds are on a single chromosome (Additional file 7, datasheet “31 Scaffolds”). On the other hand, the three isolates Pr-140.9, BS96, and 9D1 showed a copy number change only in scaffold 9. Likewise, another set of three isolates (Pr-102, Pr-218, Pr-486) showed a copy number change only in scaffold 10. These results indicate that scaffolds 9 or 10 do not physically link to any of the remaining 19 scaffolds in Fig. 6. Alternatively, only parts of the chromosomes corresponding to scaffolds 9 or 10 are aberrated e.g. forming partial trisomy (such as forming tandem duplication of chromosomal regions) in these isolates. Interestingly, various chromosomal aberrations were found in scaffold 9 among isolates from diverse host species such as trisomy in BS96 (*nwt*, bay); monosomy in Pr-140.9 (*nwt*, oak) and WSU115–0118 (*nwt*, *Rhododendron*); cnLOH in Pr-16 (*nwt*, oak), Pr-455 (*nwt*, *Osmorhiza*), and WSU107–0019 (*nwt*, *Rhododendron*) (Fig. 6). This might implicate that monosomic, cnLOH and trisomic aberrations are generated through a similar genetic mechanism such as via the formation of trisomy (3×) and subsequent breakdown to cnLOH (2×) and monosomy (1×) [27, 28].

In addition, heterozygous SNP allele analysis in combination with CNV analysis enabled inference of haplotype combinations in aneuploid and cnLOH individuals. All five trisomic isolates at scaffold 10 were found to have the identical haplotype combination, denoted as AAB. Likewise, the AAB haplotype combinations at scaffolds 5, 8, 14, 15, and 20 in isolates Pr-745#3 and Pr-472 are identical. At scaffold 9, monosomy, trisomy, and cnLOH have been identified. Interestingly, one of the two haplotypes, denoted as A, is predominant: i.e. haplotype A in monosomy, the AAB haplotype combination in trisomy, and the AA haplotype combination in cnLOH isolates (Fig. 6). Pr-16 was the only exception in which the haplotype combination at scaffold 9 is BB. Together, these observations indicate that either (1) one of the haplotypes is more readily retained in the cell or (2) the excess of the haplotype confers fitness to the environment.

## Discussion

Despite asexual propagation, isolates of *Phytophthora ramorum* display distinct colony morphological types, i.e. wild type (*wt*) and non-wild type (*nwt*). Prior work has documented that: a)- stems of mature oaks but not leaves of California bays induced phenotypic conversion from *wt* to *nwt,* and b)- the *nwt* phenotype was closely associated with the generation of aneuploidy and/or cnLOH [12]. *Nwt* phenotypes have, however, occasionally been observed in isolates from ornamental shrubs in production nurseries without the involvement of oak hosts [10]. In this report, we compared phenotypes and genomes of *nwt* isolates from nursery hosts, soil, and water baits, and those from oaks and other hosts in order to gain insights of whether the generation of *nwt* phenotype in foliar hosts and that in stem canker hosts were driven by the same genetic mechanism. Further study is necessary to determine whether there is a difference between *nwt* isolates from oaks and from other hosts and substrates, since only one oak sample was examined using both phenotypic tests and genotyping. Additionally, the availability of non-oak hosts makes the study of host-induced genomic changes much easier to accomplish, due to the difficulty of employing mature oaks in these kind of studies: oaks in fact are seriously threatened by *P. ramorum* and by other emergent pests and pathogens, and the availability of mature oaks for scientific experimentation is scarce.

Isolates with *nwt* morphology tend to grow slower [9] and show reduced aggressiveness in host plants [10, 11]. We found that two quantitative phenotypic characters of *P. ramorum*, i.e. lesion area on *Rhododendron* leaves and irregularity index of cultures, could divide isolates into three distinctive clusters using K-means analysis. All *nwt* isolates examined in this study were exclusively found in Cluster 3. In addition, all Cluster 3 isolates subjected to genome analyses were found to carry aneuploidy and/or cnLOH. These findings implicate that *nwt* colony morphology found in oak isolates and those from other hosts and substrates are phenotypically and genomically equivalent. *Wt* isolates were, on the other hand, found either in Cluster 1 or Cluster 2. Isolates in Cluster 1 were more aggressive on *Rhododendron* leaves than those in Cluster 2 but otherwise they were morphologically indistinguishable.

Because polymorphic SSR markers failed to show genetic differentiation among isolates in the clusters, change in cluster membership is likely rapid in relation to SSR evolution. As *wt* to *nwt* phenotypic conversion in oak hosts was shown to occur within 3 months [12], transition of the pathogen between K-means clusters in the foliage of ornamental plants and other substrates may also occur within a few months. Evolutionary implications of the three clusters have yet to be investigated. *Nwt* isolates from *Rhododendron* found in Cluster 3 were less aggressive in inoculation tests and reduced sporulation was observed on cultures (data not shown), suggesting that *nwt* individuals are not likely to contribute majorly to the spread of *P. ramorum*. However, in a scenario where the most aggressive genotypes kill a large number of their hosts, less aggressive genotypes may actually be longer lived and important for the long-term evolution of the species. It could also be that aneuploidy or cnLOH in *nwt* isolates may confer increased fitness under specific conditions such as in the presence of fungicides [29–31], while the same changes incur a high fitness cost in the absence of them [27]. This has been reported for aneuploid *Candida albicans* resistant to fluconazole [31, 32].

Given that aneuploidy and cnLOH occur at much higher frequency than point mutations, these changes have the potential to mediate adaptation to new environments or hosts [27]. In this research, we identified various chromosomal aberrations in isolates from diverse non-oak hosts for the first time*.* Interestingly, afflicted regions of the genomes are partly shared between trisomic, monosomic, and cnLOH aberrations, and when shared, the same haplotypes were predominant among isolates. This observation may indicate a fitness or viability advantage of the predominant haplotypes. Interestingly, cnLOH were found to be common also among isolates of the EU1 lineage of *P. ramorum* in the UK (phenotypic deviation not noted) [33]. Biological significance of the observed cnLOH is unknown. LOH has frequently been observed also in *Phytophthora capsici*, and some isolates with cnLOH displayed changes in disease phenotypes as well as mating type switching [34], indicating LOH and associated phenotype diversification is ubiquitous in oomycetes and potentially plays a major role in pathogen evolution.

In addition to cnLOH and aneuploidy, a new type of chromosomal aberration we termed short CNV (sCNV) euploid was identified in two isolates in Clusters 1 and 2. In the two sCNV euploids, the majority of CNV segments were found in the multicopy regions of the genome, such as at TEs and tandem repeats. Repeat number of TEs and tandem repeats can be changed in response to stress [35, 36] and thus have implications in adaptation to a challenging environment. Although the selective advantage of sCNV euploidy is uncertain, we should be vigilant in our scrutiny of emergence of this new type of chromosomal aberration in the pathogen population.

Diverse natural and industrial products, fungicides, antibiotics, and agricultural chemicals are known to impose stress to many of the major cellular events during cell division leading to aneuploidy [37]. For instance, common defense chemicals in plants such as coumarins and monoterpenes have been shown to cause spindle dysfunction and aneuploid formation. Derivatives of such chemicals occurring in certain host plants might be responsible for host dependency of aneuploid and cnLOH formation in *P. ramorum*. In production nurseries and landscapes, ornamentals e.g. *Rhododendron* and *Viburnum* are exposed to various agrochemicals, such as Mefenoxam. Mefenoxam is a widely used fungicide against oomycetes and has been shown to induce chromosomal aberrations in human lymphocytes [38]. In areas where such fungicides are routinely used, their use may partly explain the high incidence of chromosomal aberrations among *P. ramorum* from ornamentals in nurseries.

**Fig. 7:**
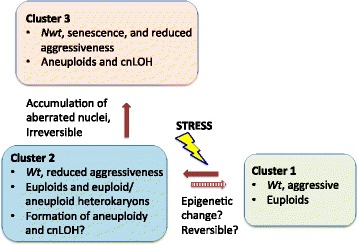
Hypothetical mechanism for host-induced phenotypic diversification and associated chromosomal alterations in *P. ramorum*. The K-means Cluster 1 (Fig. 2) is comprised of individuals that are aggressive on host foliage, showing *wt* colony type, and carrying euploid genomes (including sCNV euploid). When individuals in Cluster 1 undergo stress such as during colonizing a sub-optimal host plant, epigenetic changes occur which will cause reduction in aggressiveness. Epigenetic changes may also result in increased rate of genome aberration while growth rate and colony morphology are unaffected (Cluster 2). The transition of membership between Cluster 1 and 2 is possibly reversible. Accumulation of aberrated nuclei in the multinucleated cells will result in the manifestation of *nwt* colony type and detection of chromosomal aberrations (Cluster 3). Transition from Cluster 2 to 3 is likely irreversible

On the basis of the results described above, we formulated a model for the genome and phenotypic diversification where relationships between the three phenotypic states of *P. ramorum* revealed by K-means clustering are shown (Fig. 7). We assume that Cluster 1, in which *wt* and aggressive isolates from California and Washington State are grouped, is the basal state of the NA1 clone of *P. ramorum*. Colony morphology of isolates in Cluster 2 is indistinguishable from those in Cluster 1. Furthermore, two out of four analyzed isolates in Cluster 2 were normal euploids. In other words, euploid isolates in Cluster 1 and Cluster 2 are indistinguishable at the genome level, yet there is a significant difference in aggressiveness. Given no structural changes in the genome was detected, either point mutations or epigenetic gene regulation is the likely cause of the phenotypic differences in the clonal pathogen [39]. Because of the lack of population genetic subdivision according to the K-means clusters, transition of cluster 1 to 2 is possibly driven by epigenetics, implicating the transition is reversible. Of note, one *wt* isolate identified in Cluster 2 carried predominantly normal euploid nuclei with a small fraction of monosomic nuclei. The reduced aggressiveness is unlikely owing to the low-level mosaic aneuploidy (a small amount of aneuploid nuclei in predominantly euploid cytoplasm) as changes in gene dosages are in general attributed to detrimental phenotypes caused by aneuploidy [40]. Rather, the observation of the mosaic aneuploidy suggests genetic instability of isolates in Cluster 2, which results in an increased rate for the formation of chromosomal aberrations. Conversion from *wt* to *nwt* colony morphology is frequently observed in *P. ramorum* in the lab and an association between a high ratio of aneuploid to euploid nuclei and the *nwt* phenotype has been noted [12]. Hence, cluster 2 is likely an intermediate state between Cluster 1 and 3. It has been suggested that aneuploidy is produced by aberrant epigenetic modification of chromatin [41]. Reduced aggressiveness in Cluster 2 may also be due to epigenetic perturbation in regulatory circuits of genes required for *in planta* proliferation. Inoculation experiments have previously failed to show that *nwt* phenotype could reverse back to *wt* [12]. Transition from Cluster 2 to 3 is, therefore, likely irreversible. Further study is needed to evaluate the reversibility of transitions between the K-means clusters and the molecular basis thereof.

We have shown phenotypic changes and associated genome alterations in *P. ramorum* isolates from diverse host species. This work is pivotal for the development of a new pathosystem rather than relying on the impractical mature oak-*P. ramorum* pathosystem for the study of HIPD. In particular, use of *Rhododendron* hosts from which several isolates with genome aberrations were identified is promising for further investigation. It could also be possible that other environmental factors, such as climate in Washington State and/or fungicide applications are attributable to the observed HIPD and genome aberrations. In any case, optimization for the examination of HIPD in the *P. ramorum/ Rhododendron* pathosystem should be straightforward and the model pathosystem will open the door to study the interaction of host and invasive pathogen at genomics/epigenomics level. Judging from the frequent observation of aneuploidy, LOH, and phenotypic diversifications in several *Phytophthora* pathogens [42, 43] and the Chytrid frog pathogen [44], the phenomenon is ubiquitous and understanding the phenomenon will help us to better manage invasive pathogens.

## Conclusions

Host-induced phenotypic diversification (HIPD) is a recently described plant-pathogen interaction by which oaks but no other hosts such as California bays induce irreversible phenotypic and chromosomal alterations in the pathogen *P. ramorum*. The phenomenon is likely widespread among invasive oomycete pathogens and potentially plays a major role in pathogen evolution. The lack of practical model pathosystems has, however, hampered the study of the phenomenon. Here we compared phenotypic variation and genome alterations in clonal *P. ramorum* isolates from diverse environments and searched for an alternative pathosystem. Our results suggest that HIPD comparable to that which occurs in oak hosts also takes place in leaves of ornamental *Rhododendron*. Future studies should hence exploit the *P. ramorum*/*Rhododendron* pathosystem to investigate the genetic mechanisms underlying HIPD and roles of HIPD in pathogen evolution.

## Methods

### Isolates and culture conditions

A total of 30 isolates of *P. ramorum* in the Washington State University (WSU) culture collection were examined in this study. These isolates consisted of 23 from plants in nurseries or landscapes, four from water, and three from soil in Washington State (Table 1). All isolates belonged to the NA1 clonal lineage and were collected between 2006 and 2015. In addition, 33 isolates in the UC Davis culture collection and 40 isolates in the California Department of Food and Agriculture (CDFA) collection were used in this study, for a total of 103 isolates from all sources. Information about which isolates were used in each experiment can be found in Table 1 and the full isolate list in Additional file 1. The WSU isolates were maintained on 1/3× CV8A slants (6.6% clarified V8 juice with 1.5% Difco Bacto Agar) [45] at 15 °C until used in the study, whereas the UC Davis and CDFA isolates were maintained on small plugs of 1/3 CV8A submerged in water at 14 °C. All permits to work with *P. ramorum* in the lab and the field in California and Washington state have been secured from the California Department of Agriculture (Permit No. 2201), USDA-APHIS (Permit No. P526P-16-03679), and Washington State University (BAF# 957–004). Collecting permits have also been obtained for all sites where field plots have been established. These include California State Parks (blanket permit for all state parks), National Park Service (Redwood National Park and associated North Coast State Parks, Pt. Reyes National Seashore), Marin Municipal Water District, Monterey Regional Parks District, East Bay Regional Parks, Big Sur Land Trust, and Mid-Peninsula Open Space.

### Colony morphology and growth rate

Colony morphology and growth rate were assessed for a selected group of 34 *P. ramorum* isolates (30 WSU, 3 UC Davis, and 1 CDFA isolates) using methods in [12]. For the growth rate study, three replicate plates of each isolate were done in two trials. A 6 mm diameter inoculum plug taken from a 7 day old culture growing on 1/3× CV8A was placed mycelium side down in the center of a 6 or 9 cm Petri dish containing 1/3× CV8A. After 7 days incubation at 20 °C (colony photos in Additional file 2), colony diameters were measured in two perpendicular directions. Photographs were taken of all plates and colony morphology (irregularity index) was assessed on the plate having the most variable morphology (sectoring) per isolate from each trial.

The radial growth at 7 days for each isolate was expressed as percent deviation from 7-day radial growth of the standard wild-type isolate CDFA1418886 (abbreviated as ND886). Irregularity index, which is the maximum percent deviation of the maximum and minimum radii within a 45-degree sector of the colony (Additional file 3) was used to quantitatively assess the colony morphology.

Criteria described previously [12] were used to score colony morphology using a standard *wt* isolate: if (1) the growth rate was slower than the standard ND886 by at least 25% or (2) the irregularity index 15% or larger, these colonies were scored as “non-wild type” (*nwt*). It should be noted that colony morphology of *P. ramorum* isolates can be unstable. When *nwt* colony morphology was observed upon further subculturing, the phenotype of the isolate was described as *nwt* even though the original isolate did not consistently display *nwt* colony morphology.

### Aggressiveness assay

Relative aggressiveness of each isolate was determined by inoculating *Rhododendron* ‘Nova Zembla’ leaves with a 6 mm mycelial plug from an actively growing culture. A 5 mm wound was created at each inoculation site with a sterile scalpel. Five replicate *Rhododendron* ‘Nova Zembla’ leaves were inoculated with each isolate and two trials were conducted. Lesion area was measured using the program APS ASSESS V 2.0 (Lamari, 2008) after 10 days of incubation at 20 °C in a moist chamber. Aggressiveness for each isolate was expressed as percent deviation of lesion area from lesion area of the standard wild-type isolate ND886.

### Data analysis for phenotype tests

All variables were examined for normality using the Shapiro-Wilk test and trials for homogeneity of variance using Levene’s test. Since the result for Levene’s test was not significant for any of the variables, the trials were pooled. Descriptive statistics of percent deviation of lesion area and growth rate, and of colony irregularity index from reference wild-type isolate ND886 were calculated. The optimal number of clusters for K-means was determined using the gap statistic [18] and found to be 3. K-means cluster analysis was done on standardized mean values of these three variables using the packages cluster [46] and factoextra [47] in the statistical software R v.3.4.0 [48]

### Log inoculation and re-isolation

The log inoculation technique has been used to assess disease susceptibility to *Phytophthora* species [49]. We use the method to evaluate host-induced phenotypic diversification in three oak species: i.e. interior live oak (*Quercus wislizenii*), coast live oak (*Quercus agrifolia*)*,* and Engelmann oak (*Quercus engelmannii*). Logs were cut from three individual mature trees per species grown in Cleveland National Forest and Mt. San Jacinto State Park, California in June 2012. The bottom and top ends of the logs were immediately sealed with a water-based emulsion sealant (Waxlor, Willamette Valley, Co., Eugene, Oregon). Holes 6 mm in diameter were made to the cambial zone of logs with a cork borer and inoculation was made with a 6 mm diameter agar plug cut from the margin of a 7-day old culture growing on 1/3 V8A (6.6% non-clarified V8 with 1.5% agar) and the openings were sealed with Parafilm and incubated at 20 °C. Thirty-six days after inoculation, cankers that developed under tree bark were exposed and small pieces of phloem tissue cut from canker margins were placed onto PARP selective medium [50] for re-isolation. A total of 259 re-isolates were established and colony morphology was scored. Two, five, and three re-isolates from interior live oak, coast live oak, and Engelmann oak, respectively, were subjected to CNV analysis. In addition, CNV data from previously published work [11] on three re-isolates from canyon live oak (*Q. chrysolepis*) were included in this analysis.

### SSR multilocus genotype analysis

The 30 Washington isolates and the standard California NA1 isolate (ND886) were transferred from archive to PARP-V8 medium and then to cellophane overlaid on PARP-V8. After 5 days of growth, the mycelium was scraped from the surface of the cellophane and into a DNA extraction tube with six 3 mm glass beads and temporarily frozen at − 80 °C. Frozen samples were homogenized with a FastPrep homogenizer (MP Biomedicals) and DNA was isolated using the Gentra Puregene Tissue Kit (Qiagen), then quantitated and normalized.

Eleven published microsatellite markers [51–53] were amplified in three multiplex reactions. Locus 82 was eliminated due to inconsistent results and ILVOPrMS79 and ILVOPrMS145 were eliminated due to unclear data, leaving eight microsatellites in the final analysis (Additional file 5). Base-pair sizes at each of the eight SSR loci were determined from raw fragment-analysis data using GeneMapper v4.0 software (Applied Biosystems) with the Microsatellite Default Analysis Method. The alleles were then called based on base-pair sizes and calibration with nine samples within this set that were previously analyzed by another lab. Allele calls were confirmed by the second analysis. Data were formatted in GenAlEx format (Excel) and imported into the R statistical program [48]. To visualize the relationships among multilocus genotypes (MLGs), minimum spanning network based on Bruvo’s distance [54] was constructed using the package Poppr 2.2.0 [55]. AMOVA was used to determine the presence of significant genetic structuring among the identified K-means clusters using Poppr 2.2.0.

### DNA extraction for Illumina DNA sequencing

Thirty-two isolates (8 from Washington State, WA 2017 and 24 from California, CA 2017 in Table 1) were selected for Illumina DNA sequencing, and data from previously published sequences of ten isolates was included for comparative purposes [11] (UC Davis Collection, genome analysis published, CA 2016 in Table 1). Eleven of these isolates (3 from California and 8 from Washington State) were also used in the phenotype testing described above (Table 1). Details of DNA extraction and Illumina DNA sequencing can be found elsewhere [12]. In essence, each *P. ramorum* isolate was grown on 1× CV8A overlaid with a polycarbonate membrane filter (catalog no. 28157–927; VWR) for 7 days at 21 °C in dark. Each circular mycelial mat was then peeled off and immediately snap-frozen in liquid nitrogen and kept at − 70 °C until use. Frozen tissues were chilled in liquid nitrogen, pulverized using mortar and pestle, and subsequently genomic DNA was extracted according to the User-Developed Protocol for filamentous fungi using the Qiagen Genomic-tip 20/G [56]. This method yielded up to 10 μg of genomic DNA. Paired-end libraries were constructed according to the manufacturer’s instructions for TruSeq DNA PCR-Free Library Preparation Kit (Illumina, Inc).

### DNA-seq data analysis

The data analysis pipeline can be found elsewhere [12] with a few changes. Briefly, the processed Illumina reads were aligned to the reference genome of *P. ramorum* isolate ND886 (URL www.eumicrobedb.org/ ND886 V1) using Burrow-Wheeler Aligner (BWA) [57] with default parameters. Two methods were used to evaluate copy number variation (CNV) in each of *P. ramorum* isolates. The first method detects CNVs from BWA aligned reads using a read-depth algorithm called BIC-seq [58]. To minimize experimental noise, the genome sequence isolate ND886 (CDFA1418886), an euploid isolate from *Camellia* found in a nursery, and Pr-1556, an euploid isolate from California bay, were included in every sequencing run and used to estimate CNVs in the samples processed and run on the Illumina genome sequencer at the same time. The second method infers CNV from read-depth ratios of alleles at heterozygous sites. SAMtools was used to process aligned reads [59] and heterozygous sites or single nucleotide polymorphisms (SNPs) were identified from the aligned reads using Bcftools [60]. An average ratio of reads of heterozygous alleles in sliding non-overlapping windows of 10 Kb across each scaffold was then used to infer CCNVs [44]. Loss of heterozygosity (LOH) was also inferred from read-depth ratios of alleles at heterozygous sites. This method is identical to the second CCNV analysis mentioned above but any allele ratios equal or larger than eight were set to 8.

### Haplotype estimation of scaffolds

Haplotypes of 19 trisomic, monosomic and cnLOH isolates were inferred from ratios or presence/absence of heterozygous loci at contigs showing CCNVs (Additional file 7 datasheet “31 Scaffolds”). Aligned reads were examined in IGV- the Integrative Genomics Viewer (http://software.broadinstitute.org/software/igv/) [61, 62] and heterozygous SNP markers located near the left border, center, and right border of each scaffold were used to genotype each isolate. Genomic regions covered by Illumina reads mapped to multiple locations were masked for SNP marker selection. SNP genotypes of isolates showing CCNVs were presented in Additional file 7 datasheet “Fig. 6 SNPs”. Haplotype combinations of isolates at each scaffold were inferred using heterozygous SNP marker genotypes together with the ratio of coverage of each of alleles at each locus.

### Identification of repeats in the *P. ramorum* genome

Prediction of transposable elements was performed by TransposonPSI, which use a PSI-blast search of a nucleotide sequence against a set of profiles of proteins corresponding to major clades/families of transposon open reading frame (http://transposonpsi.sourceforge.net/).

Tandem Repeat Finder (https://tandem.bu.edu/trf/trf.html) [63] was used to locate tandem repeats in the *P. ramorum* genome. Of the 9954 tandem repeats, 1578 repeats, which were equal or large than 100 bp (which is equal to the minimum CNV output of BIC-seq analysis), were used for the characterization of CNV regions.

Multicopy regions in the genome were indicated in the Sequence Alignment/Map (SAM) file. Illumina reads of isolate ND886 was aligned to the *P. ramorum* genome using Burrow-Wheeler Aligner (BWA) [57] and a SAM was generated. The tag X0 in the SAM file specifies the number of best hits of an Illumina read in the genome. An average X0 numbers of reads in sliding non-overlapping windows of 1000 bp across each scaffold was calculated and a genome segment with average X0 > 2 is defined as a multicopy region.

## Additional files

Additional file 1: Details of NA1 *P. ramorum* isolates used in this study. Other names, source, geographical location, year of isolation, SSR multilocus genotypes, colony phenotype, relative radial growth rate, relative lesion size, irregularity index, K-means cluster, CCNV type and contact scientists for isolates are shown. (XLSX 19 kb)

Additional file 2: Colony morphology of Washington State isolates seen on Petri plates. *Nwt* phenotype is indicated. Colonies were grown on solid 1× CV8A medium for 7 days at 20 °C in dark. WSU115–0077 initially showed *wt* colony morphology, however, it displayed *nwt* in subsequent subculture. (PDF 1200 kb)

Additional file 3: The maximum and minimum radii within a 45-degree sector of the colony were used to obtain irregularity index as: (43.5–34.7)/43.5 = 20.8% (PDF 960 kb)

Additional file 4: Mean values for irregularity index and relative lesion area of *Phytophthora ramorum* isolates identified in K-means analysis. Isolates in Cluster 3 were significantly different from those in Clusters 1 and 2. Bars with different letters are significantly different at *p* < 0.001 (One-way ANOVA, Tukey-Kramer multiple comparisons). (PDF 41 kb)

Additional file 5: Allele sizes for the 15 multilocus genotypes (MLGs) identified in 30 Washington State isolates of *P. ramorum*. (PDF 48 kb)

Additional file 6: Diverse CCNVs revealed by BIC-seq analysis (upper graph for each panel) and a read-depth analysis for heterozygous allele ratios using 10 Kb long non-overlapping sliding window (lower graph). A concatenated view of the 30 largest scaffolds with the total length of 30 MB, which corresponding to approximately a half of the total genome of *Phytophthora ramorum*, are shown. Scaffolds numbers for large CCNV regions are indicated with pink bars, and those for copy number neutral LOH are shown with blue bars. Scales show log (base 2) fold difference between sample isolates and reference isolates for BIC-seq analysis and log (base 2) ratios of alleles of sample isolates for the heterozygous allele ratio analysis. At each heterozygous locus, a read count ratio (more-abundant allele/less-abundant allele) was calculated. A) A re-isolate 9D1 from a log of coast live oak showing a small copy number change at scaffold 9 in comparison to its original isolate Pr-1556 (*wt*, bay) used as a reference. 9D1 is likely a heterokaryon (HK) carrying trisomic (3× CCNV) and euploid nuclei. B) Pr-16 carries trisomy and cnLOH aberrations. C) MK516a carries cnLOH. D) BS2014–584 shows a complicated patter indicating it carries a mixture of trisomic and monosomic nuclei. E) Pr-140.7 is a complicated monosomic heterokaryon. F) Pr-140.9 is monosomic at scaffold 9. G) Pr-106 is a normal euploid. H) Pr-486 is a trisomy at scaffold 10. Numbers of short segmental CNVs are also seen. I) and J) Both re-isolates Pr-745#4 and Pr-1556#7#1 carry complicated mixtures of monosomic and trisomic nuclei. K) BS96 from California bay is trisomic at scaffold 9. L) Pr-218 from *Rhamnus cathartica* is trisomic at scaffold 10. M) Pr-102, the sequence isolate (Tyler et al..., [17]) is trisomic at scaffold 10. N) a re-isolate Pr-745#3 is trisomic. O) Pr-455 from *Osmorhiza berteroi* shows cnLOH at several scaffolds. P) Pr-472 from *Choisya ternate* is trisomic. (PDF 3465 kb)

Additional file 7: Details of physical linkage of genome scaffolds and inferred haplotype combinations at each scaffold. Two datasheets are in the file. Datasheet “31 Scaffolds”: CCNVs found in the largest 31 scaffolds are shown. A and B denote homologous haplotypes at each scaffold. Inferred haplotype combinations are shown when those are deviated from a heterozygous disomic haplotype combination, AB. AAB, AA, and A denote trisomy, cnLOH, and monosomy, respectively. AB + XXX indicates a mixture of AB disomy and trisomy with an unknown haplotype combination. HK in CCNV type indicates a heterokaryon. Scaffolds 5, 8, 14, 15, and 20 are likely physically linked, whereas each of scaffolds 9 and 10 do not link to any of the 31 largest scaffolds in the current assembly. Datasheet “Fig. 6 SNPs”: Three representative SNPs located near the left border, center and right border of each scaffold in combination with read coverage were used to infer haplotype combinations presented in Fig. 6. (XLSX 23 kb)

## Abbreviations

AMOVA: Analysis of molecular variance
CA: State of California
CCNV: Chromosomal copy number variation
cnLOH: chromosomal number neutral loss of heterozygosity
CNV: Copy number variation
HIPD: Host-induced phenotypic diversification
LOH: Loss of heterozygosity
MLG: Multilocus genotype
*nwt*: non-wild type
sCNV euploid: small copy number variation euploid
SNP: Single nucleotide polymorphism
SSR: Simple sequence repeat
TE: Transposable element
WA: State of Washington
*wt*: wild type
