# Supplementary material for: Characterization of phenotypic variation and genome aberrations observed among Phytophthora ramorum isolates from diverse hosts
Source: BMC Genomics. 2018 May 2;19:320. doi: 10.1186/s12864-018-4709-7 (PMC5932867; doi:10.1186/s12864-018-4709-7)
Supplement: Supplementary file 2 — Colony morphology of Washington State isolates seen on Petri plates. Nwt phenotype is indicated. Colonies were grown on solid 1× CV8A medium for 7 days at 20 °C in dark. WSU115–0077 initially showed wt colony morphology, however, it displayed nwt in subsequent subculture. (PDF 1200 kb) [file 12864_2018_4709_MOESM2_ESM.pdf]

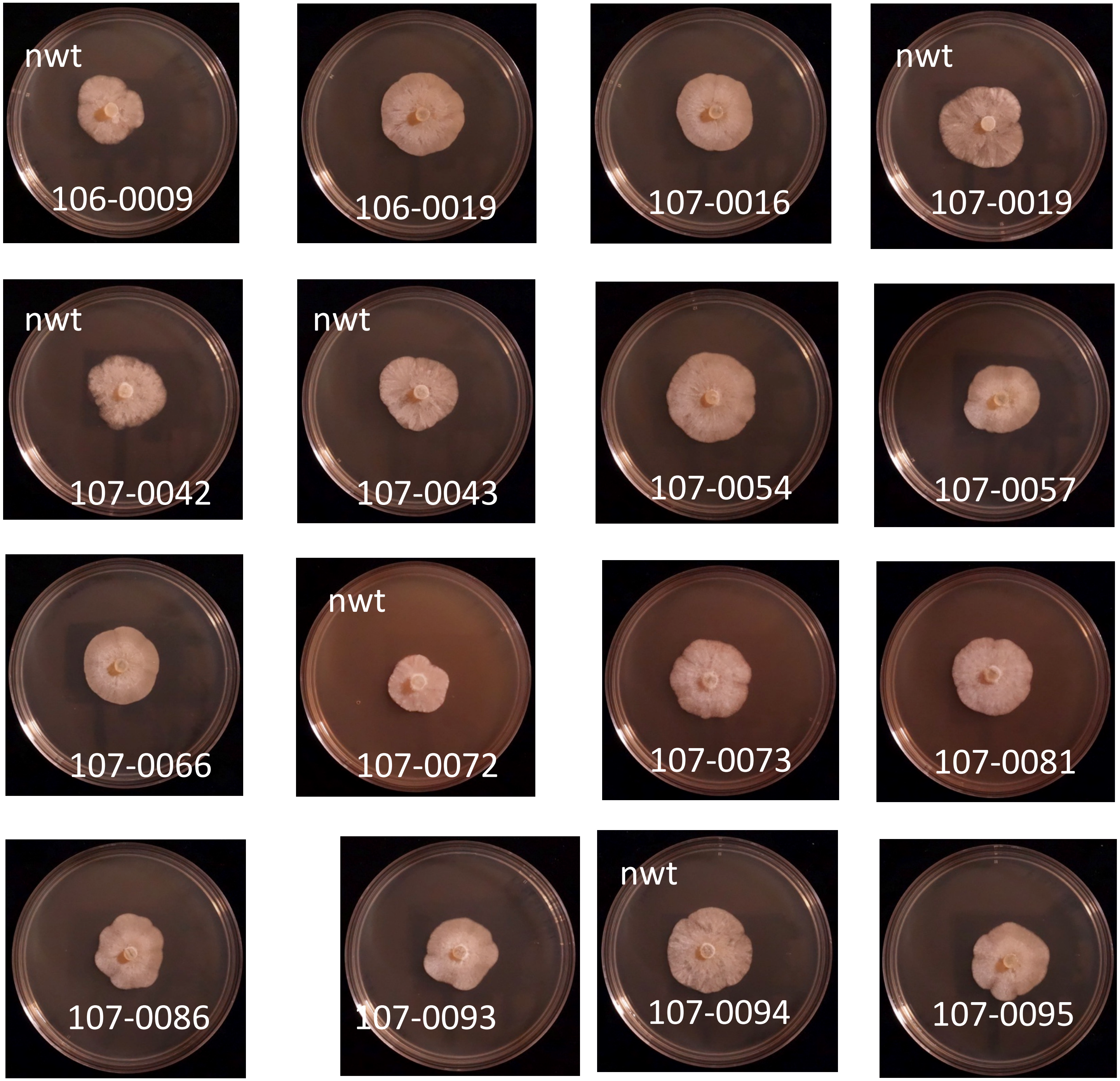
nwt
106-0009
106-0019
107-0016
nwt
107-0019
nwt
107-0042
nwt
107-0043
107-0054
107-0057
107-0066
nwt
107-0072
107-0073
107-0081
107-0086
107-0093
nwt
107-0094
107-0095

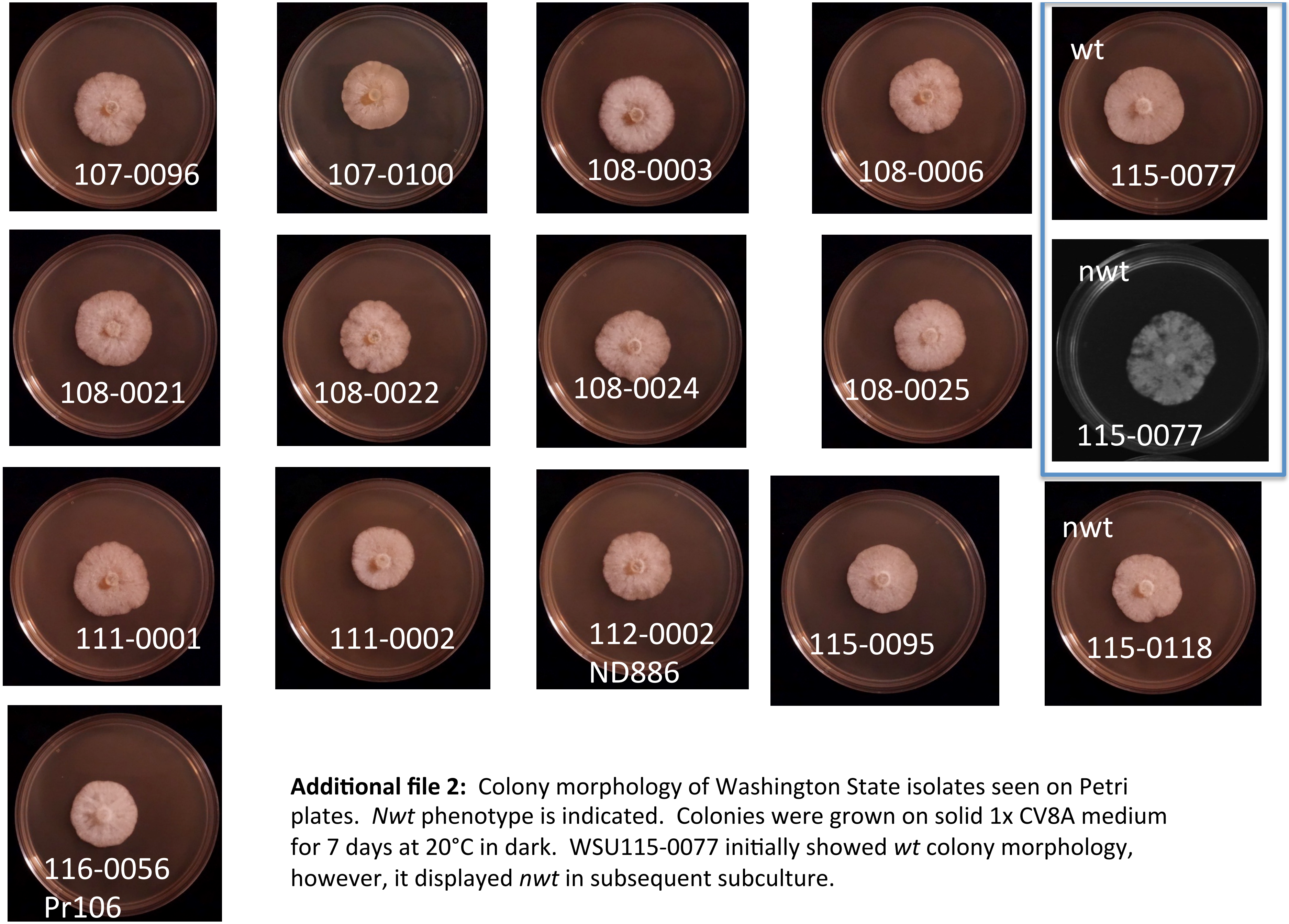

Additional file 2: Colony morphology of Washington State isolates seen on Petri plates. *Nwt* phenotype is indicated. Colonies were grown on solid 1x CV8A medium for 7 days at 20°C in dark. WSU115-0077 initially showed *wt* colony morphology, however, it displayed *nwt* in subsequent subculture.
