# Supplementary material for: Characterization of phenotypic variation and genome aberrations observed among Phytophthora ramorum isolates from diverse hosts
Source: BMC Genomics. 2018 May 2;19:320. doi: 10.1186/s12864-018-4709-7 (PMC5932867; doi:10.1186/s12864-018-4709-7)

Additional file 3. The maximum and minimum radii within a 45-degree sector of the colony is used to obtain irregularity index as: (43.5-34.7)/43.5 = 0.21

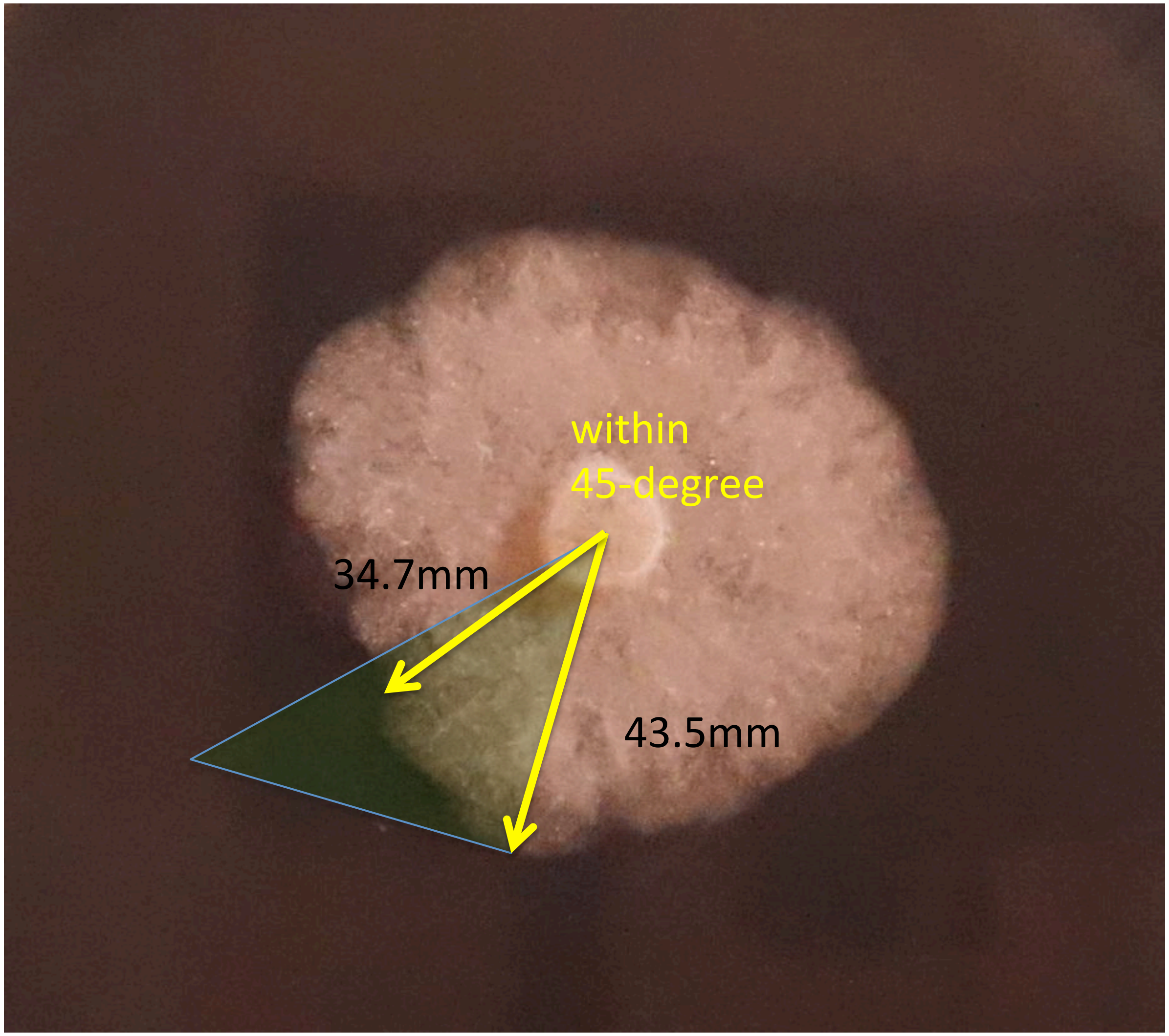

Supplement: Supplementary file 3 — The maximum and minimum radii within a 45-degree sector of the colony were used to obtain irregularity index as: (43.5–34.7)/43.5 = 20.8% (PDF 960 kb) [file 12864_2018_4709_MOESM3_ESM.pdf]
