# Supplementary material for: Characterization of phenotypic variation and genome aberrations observed among Phytophthora ramorum isolates from diverse hosts
Source: BMC Genomics. 2018 May 2;19:320. doi: 10.1186/s12864-018-4709-7 (PMC5932867; doi:10.1186/s12864-018-4709-7)
Supplement: Supplementary file 4 — Mean values for irregularity index and relative lesion area of Phytophthora ramorum isolates identified in K-means analysis. Isolates in Cluster 3 were significantly different from those in Clusters 1 and 2. Bars with different letters are significantly different at p < 0.001 (One-way ANOVA, Tukey-Kramer multiple comparisons). (PDF 41 kb) [file 12864_2018_4709_MOESM4_ESM.pdf]

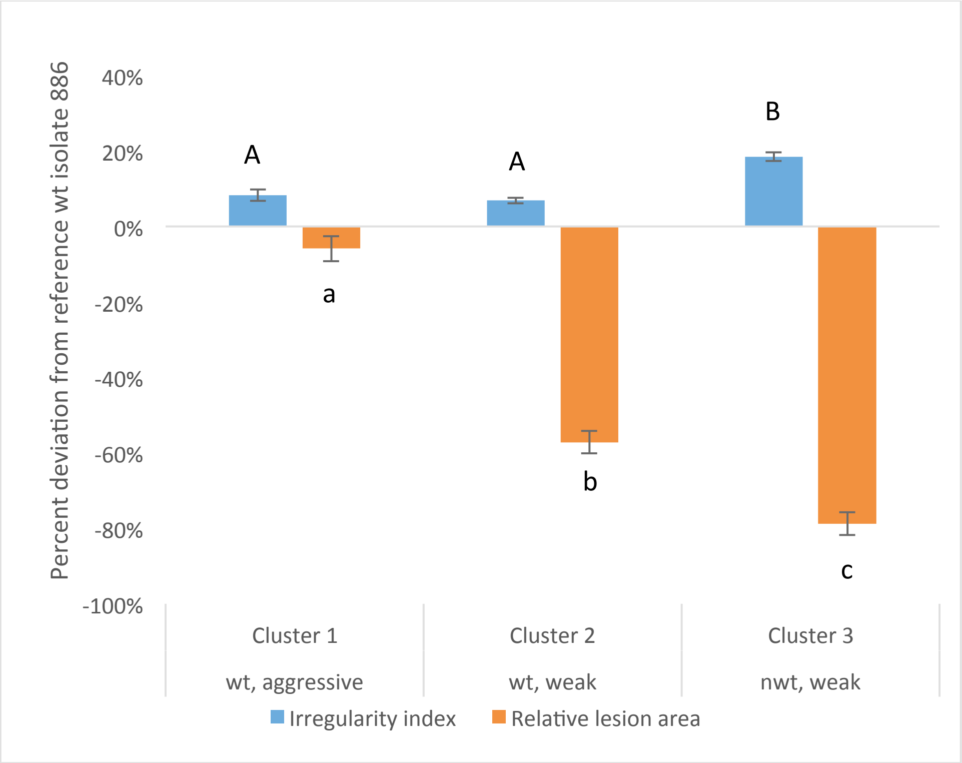

Additional file 4: Mean values for irregularity index and relative lesion area of *Phytophthora ramorum* isolates identified in K-means analysis. Isolates in Cluster 3 were significantly different from those in Clusters 1 and 2. Bars with different letters are significantly different at $p < 0.001$ (One-way ANOVA, Tukey-Kramer multiple comparisons).
