# Supplementary material for: Characterization of phenotypic variation and genome aberrations observed among Phytophthora ramorum isolates from diverse hosts
Source: BMC Genomics. 2018 May 2;19:320. doi: 10.1186/s12864-018-4709-7 (PMC5932867; doi:10.1186/s12864-018-4709-7)
Supplement: Supplementary file 5 — Allele sizes for the 15 multilocus genotypes (MLGs) identified in 30 Washington State isolates of P. ramorum. (PDF 48 kb) [file 12864_2018_4709_MOESM5_ESM.pdf]

Additional file 5. Allele sizes for the 15 multilocus genotypes (MLGs) identified in 30 Washington State isolates of *P. ramorum*.

Isolate	SSR MLG	Cluster in Fig. 2	PrMS6*	Pr9C3*	PrMS39*	PrMS45*	PrMS43*	Locus18**	Locus64**	ILVOPrMS131***
WSU106_0009	NA1-D02	3	165/168	216/226	254/254	166/186	368/485	219/275	342/379	150/228
WSU106_0019	NA1-D06	2	165/168	216/226	254/254	166/186	396/481	219/275	342/379	150/228
WSU107_0016	NA1-D01	1	165/168	216/226	254/254	166/186	372/485	219/275	342/379	150/228
WSU107_0019	NA1-A21	3	165/168	216/226	250/250	166/0 #	364/485	219/275	342/379	150/232
WSU107_0042	NA1-A21	3	165/168	216/226	250/250	166/186	364/485	219/275	342/379	150/232
WSU107_0043	NA1-A21	3	165/168	216/226	250/250	166/186	364/485	219/275	342/379	150/232
WSU107_0054	NA1-A03	2	165/168	216/226	250/250	166/186	368/485	219/275	342/379	150/228
WSU107_0057	NA1-A03	1	165/168	216/226	250/250	166/186	368/485	219/275	342/379	150/228
WSU107_0066	NA1-D01	1	165/168	216/226	254/254	166/186	372/485	219/275	342/379	150/228
WSU107_0072	NA1-A05	3	165/168	216/226	250/250	166/186	368/489	219/275	342/379	150/228
WSU107_0073	NA1-A05	2	165/168	216/226	250/250	166/186	368/489	219/275	342/379	150/228
WSU107_0081	NA1-A25	2	165/168	216/226	250/250	166/186	368/489	219/275	342/379	150/232
WSU107_0086	NA1-A03	1	165/168	216/226	250/250	166/186	368/485	219/275	342/379	150/228
WSU107_0093	NA1-D02	1	165/168	216/226	254/254	166/186	368/485	219/275	342/379	150/228
WSU107_0094	NA1-A03	3	165/168	216/226	250/250	166/186	368/485	219/275	342/379	150/228
WSU107_0095	NA1-A10	1	165/168	216/226	250/250	166/186	368/481	219/275	342/379	150/228
WSU107_0096	NA1-A10	2	165/168	216/226	250/250	166/186	368/481	219/275	342/379	150/228
WSU107_0100	NA1-A29	2	165/168	216/226	250/250	166/186	372/485	219/275	342/379	150/228
WSU108_0003	NA1-A28	2	165/168	216/226	250/250	166/186	364/485	219/277	342/379	150/224
WSU108_0006	NA1-A17	1	165/168	216/226	250/250	166/186	356/485	219/275	342/379	150/228
WSU108_0021	NA1-A02	2	165/168	216/226	250/250	166/186	364/485	219/275	342/379	150/228
WSU108_0022	NA1-A02	2	165/168	216/226	250/250	166/186	364/485	219/275	342/379	150/228
WSU108_0024	NA1-A02	2	165/168	216/226	250/250	166/186	364/485	219/275	342/379	150/228
WSU108_0025	NA1-A02	1	165/168	216/226	250/250	166/186	364/485	219/275	342/379	150/228
WSU111_0001	NA1-A29	2	165/168	216/226	250/250	166/186	372/485	219/275	342/379	150/228
WSU111_0002	NA1-A05	1	165/168	216/226	250/250	166/186	368/489	219/275	342/379	150/228
WSU115_0077	NA1-A13	3	165/168	216/226	250/250	166/186	376/485	219/275	342/379	150/228
WSU115_0089	NA1-A17	2	165/168	216/226	250/250	166/186	356/485	219/275	342/379	150/228
WSU115_0095	NA1-A30	1	165/168	216/226	250/250	166/186	356/489	219/275	342/379	150/228
WSU115_0118	NA1-A13	3	165/168	216/226	250/250	166/186	376/485	219/275	342/379	150/228

* Prospero et al., 2007
** Ivors et al., 2006
*** Vercauteren et al., 2011
# Absence of the alternative allele due to cnLOH at scaffold 9.
