# Supplementary material for: Characterization of phenotypic variation and genome aberrations observed among Phytophthora ramorum isolates from diverse hosts
Source: BMC Genomics. 2018 May 2;19:320. doi: 10.1186/s12864-018-4709-7 (PMC5932867; doi:10.1186/s12864-018-4709-7)
Supplement: Supplementary file 6 — Diverse CCNVs revealed by BIC-seq analysis (upper graph for each panel) and a read-depth analysis for heterozygous allele ratios using 10 Kb long non-overlapping sliding window (lower graph). A concatenated view of the 30 largest scaffolds with the total length of 30 MB, which corresponding to approximately a half of the total genome of Phytophthora ramorum, are shown. Scaffolds numbers for large CCNV regions are indicated with pink bars, and those for copy number neutral LOH are shown with blue bars. Scales show log (base 2) fold difference between sample isolates and reference isolates for BIC-seq analysis and log (base 2) ratios of alleles of sample isolates for the heterozygous allele ratio analysis. At each heterozygous locus, a read count ratio (more-abundant allele/less-abundant allele) was calculated. A) A re-isolate 9D1 from a log of coast live oak showing a small copy number change at scaffold 9 in comparison to its original isolate Pr-1556 (wt, bay) used as a reference. 9D1 is likely a heterokaryon (HK) carrying trisomic (3× CCNV) and euploid nuclei. B) Pr-16 carries trisomy and cnLOH aberrations. C) MK516a carries cnLOH. D) BS2014–584 shows a complicated patter indicating it carries a mixture of trisomic and monosomic nuclei. E) Pr-140.7 is a complicated monosomic heterokaryon. F) Pr-140.9 is monosomic at scaffold 9. G) Pr-106 is a normal euploid. H) Pr-486 is a trisomy at scaffold 10. Numbers of short segmental CNVs are also seen. I) and J) Both re-isolates Pr-745#4 and Pr-1556#7#1 carry complicated mixtures of monosomic and trisomic nuclei. K) BS96 from California bay is trisomic at scaffold 9. L) Pr-218 from Rhamnus cathartica is trisomic at scaffold 10. M) Pr-102, the sequence isolate (Tyler et al..., [17]) is trisomic at scaffold 10. N) a re-isolate Pr-745#3 is trisomic. O) Pr-455 from Osmorhiza berteroi shows cnLOH at several scaffolds. P) Pr-472 from Choisya ternate is trisomic. (PDF 3465 kb) [file 12864_2018_4709_MOESM6_ESM.pdf]

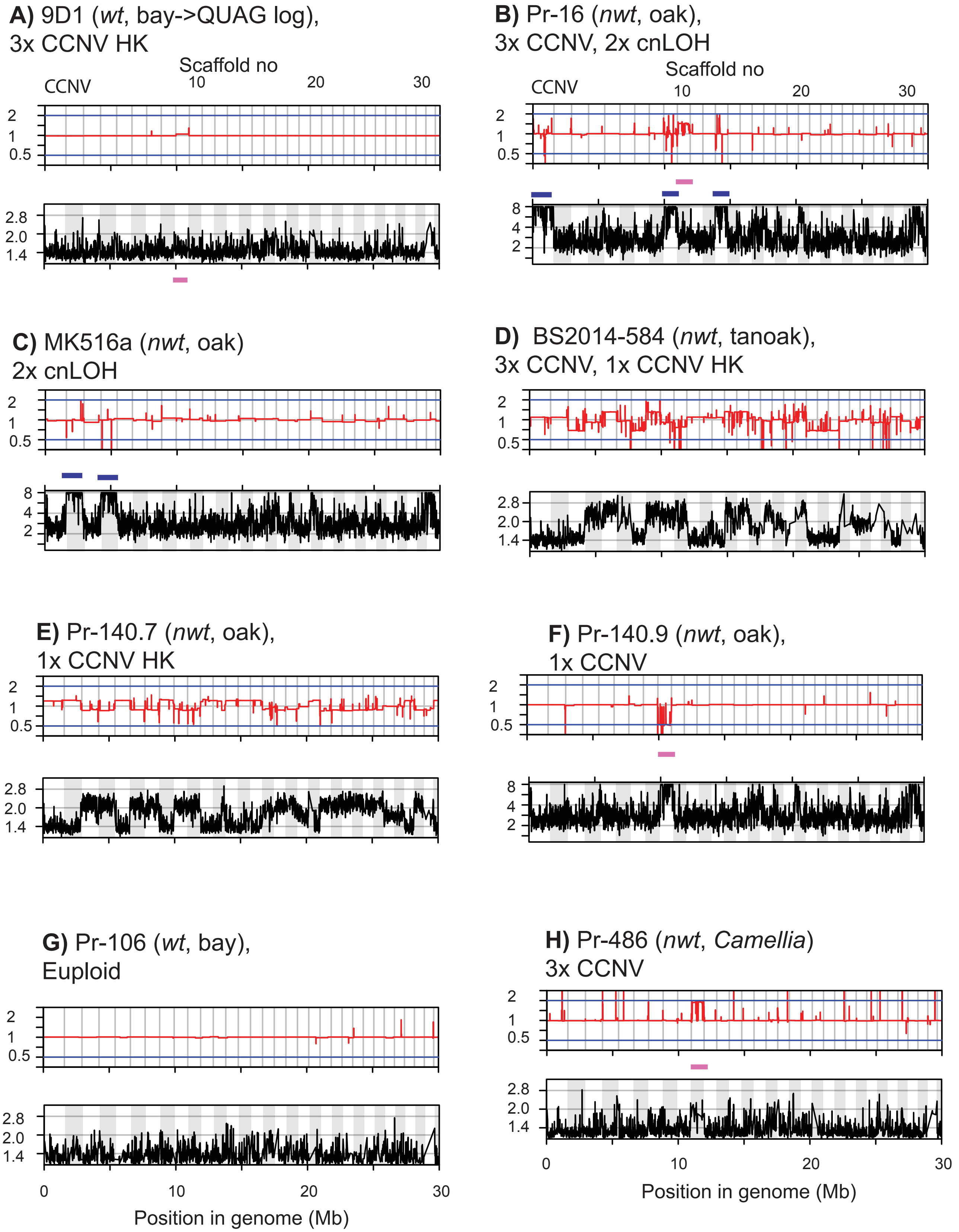
A) 9D1 (wt, bay->QUAG log),
3x CCNV HK
Scaffold no
CCNV
10
20
30
2
1
0.5
2.8
2.0
1.4
B) Pr-16 (nwt, oak),
3x CCNV, 2x cnLOH
Scaffold no
CCNV
10
20
30
8
4
2
C) MK516a (nwt, oak)
2x cnLOH
D) BS2014-584 (nwt, tanoak),
3x CCNV, 1x CCNV HK
E) Pr-140.7 (nwt, oak),
1x CCNV HK
F) Pr-140.9 (nwt, oak),
1x CCNV
G) Pr-106 (wt, bay),
Euploid
H) Pr-486 (nwt, Camellia)
3x CCNV
0
10
20
30
Position in genome (Mb)
Position in genome (Mb)

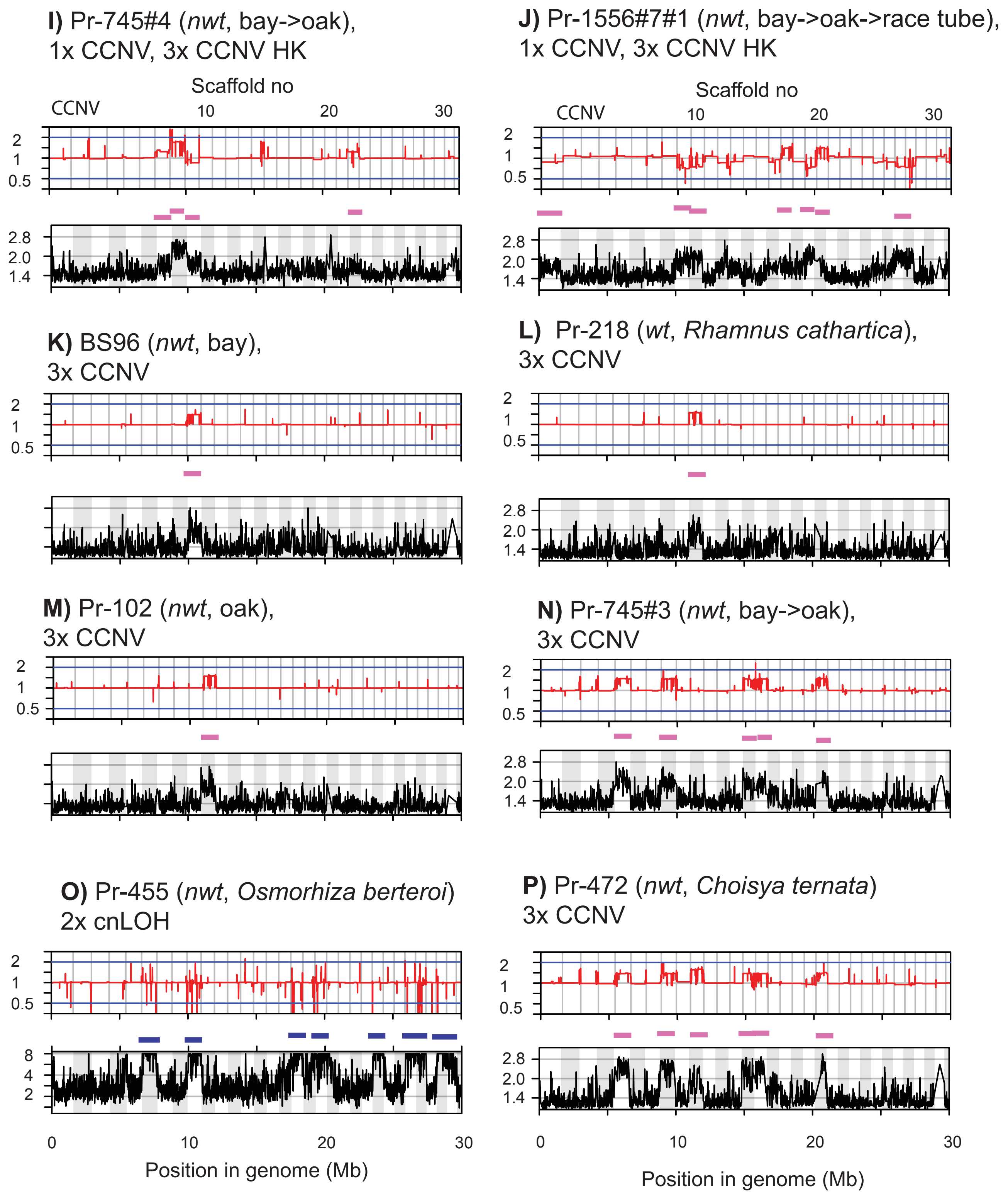
I) Pr-745#4 (*nwt*, bay->oak),
1x CCNV, 3x CCNV HK
Scaffold no
CCNV
10
20
30
2
1
0.5
2.8
2.0
1.4
J) Pr-1556#7#1 (*nwt*, bay->oak->race tube),
1x CCNV, 3x CCNV HK
Scaffold no
CCNV
10
20
30
2
1
0.5
2.8
2.0
1.4
K) BS96 (*nwt*, bay),
3x CCNV
2
1
0.5
L) Pr-218 (*wt*, *Rhamnus cathartica*),
3x CCNV
2
1
0.5
2.8
2.0
1.4
M) Pr-102 (*nwt*, oak),
3x CCNV
2
1
0.5
N) Pr-745#3 (*nwt*, bay->oak),
3x CCNV
2
1
0.5
2.8
2.0
1.4
O) Pr-455 (*nwt*, *Osmorhiza berteroi*)
2x cnLOH
2
1
0.5
8
4
2
0
10
20
30
Position in genome (Mb)
P) Pr-472 (*nwt*, *Choisya ternata*)
3x CCNV
2
1
0.5
2.8
2.0
1.4
0
10
20
30
Position in genome (Mb)

Additional file 6: Diverse CCNVs revealed by BIC-seq analysis (upper graph for each panel) and a read-depth analysis for heterozygous allele ratios using 10 Kb long non-overlapping sliding window (lower graph). A concatenated view of the 30 largest scaffolds with the total length of 30 MB, which corresponding to approximately a half of the total genome of *Phytophthora ramorum*, are shown. Scaffolds numbers for large CCNV regions are indicated with pink bars, and those for copy number neutral LOH are shown with blue bars. Scales show log (base 2) fold difference between sample isolates and reference isolates for BIC-seq analysis and log (base 2) ratios of alleles of sample isolates for the heterozygous allele ratio analysis. At each heterozygous locus, a read count ratio (more-abundant allele/less-abundant allele) was calculated. A) A re-isolate 9D1 from a log of coast live oak showing a small copy number change at scaffold 9 in comparison to its original isolate Pr-1556 (*wt*, bay) used as a reference. 9D1 is likely a heterokaryon (HK) carrying trisomic (3x CCNV) and euploid nuclei. B) Pr-16 carries trisomy and cnLOH aberrations. C) MK516a carries cnLOH. D) BS2014-584 shows a complicated patter indicating it carries a mixture of trisomic and monosomic nuclei. E) Pr-140.7 is a complicated monosomic heterokaryon. F) Pr-140.9 is monosomic at scaffold 9. G) Pr-106 is a normal euploid. H) Pr-486 is a trisomy at scaffold 10. Numbers of short segmental CNVs are also seen. I) and J) Both re-isolates Pr-745#4 and Pr-1556#7#1 carry complicated mixtures of monosomic and trisomic nuclei. K) BS96 from California bay is trisomic at scaffold 9. L) Pr-218 from *Rhamnus cathartica* is trisomic at scaffold 10. M) Pr-102, the sequence isolate (Tyler et al., 2006) is trisomic at scaffold 10. N) a re-isolate Pr-745#3 is trisomic. O) Pr-455 from *Osmorhiza berteroi* shows cnLOH at several scaffolds. P) Pr-472 from *Choisya ternate* is trisomic.
